# Wheel‐Running Exercise Alleviates Anxiety‐Like Behavior via Down‐Regulating S‐Nitrosylation of Gephyrin in the Basolateral Amygdala of Male Rats

**DOI:** 10.1002/advs.202400205

**Published:** 2024-07-04

**Authors:** Ping‐Fen Yang, Tai‐Lei Nie, Xia‐Nan Sun, Lan‐Xin Xu, Cong Ma, Fang Wang, Li‐Hong Long, Jian‐Guo Chen

**Affiliations:** ^1^ State Key Laboratory for Diagnosis and Treatment of Severe Zoonotic Infectious Diseases Department of Pharmacology School of Basic Medicine Tongji Medical College Huazhong University of Science and Technology Wuhan 430030 China; ^2^ The Key Laboratory for Drug Target Researches and Pharmacodynamic Evaluation of Hubei Province Wuhan 430030 China; ^3^ Key Laboratory of Molecular Biophysics of the Ministry of Education College of Life Science and Technology Huazhong University of Science and Technology Wuhan 430030 China; ^4^ Hubei Shizhen Laboratory Wuhan 430030 China

**Keywords:** anxiety, GABA_A_R, SNO‐gephyrin, the basolateral amygdala, wheel‐running exercise

## Abstract

Physical exercise has beneficial effect on anxiety disorders, but the underlying molecular mechanism remains largely unknown. Here, it is demonstrated that physical exercise can downregulate the S‐nitrosylation of gephyrin (SNO‐gephyrin) in the basolateral amygdala (BLA) to exert anxiolytic effects. It is found that the level of SNO‐gephyrin is significantly increased in the BLA of high‐anxiety rats and a downregulation of SNO‐gephyrin at cysteines 212 and 284 produced anxiolytic effect. Mechanistically, inhibition of SNO‐gephyrin by either Cys212 or Cys284 mutations increased the surface expression of GABA_A_R γ2 and the subsequent GABAergic neurotransmission, exerting anxiolytic effect in male rats. On the other side, overexpression of neuronal nitric oxide synthase in the BLA abolished the anxiolytic‐like effects of physical exercise. This study reveals a key role of downregulating SNO‐gephyrin in the anxiolytic effects of physical exercise, providing a new explanation for protein post‐translational modifications in the brain after exercise.

## Graphical abstract

This study explored the molecular mechanism underlay the therapeutic effect of physical exercise on anxiety disorders. Physical exercise can downregulate the S‐nitrosylation of gephyrin (SNO‐gephyrin) in the basolateral amygdala (BLA) and increase the surface expression of GABA_A_R γ2 and the subsequent GABAergic neurotransmission, exerting anxiolytic effect.

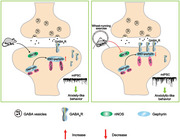

## 1 Introduction

Anxiety disorders are among the most common mental illnesses, with a prevalence of ≈25% population worldwide.^[^ ^1^ ^]^ Lifetime prevalence of anxiety disorders has been estimated to be between 15% and 20%.^[^ ^2^ ^]^ The symptoms of anxiety disorders are complex and varying, however, the exact neural mechanisms are still poorly understood. Currently, psychotherapy (especially cognitive‐behavioral therapy) and drugs (especially serotonergic compounds) are used to treat anxiety disorders.^[^ ^3^ ^]^ However, long‐term use of these drugs is easy to produce drug dependence, and the clinical needs are still unresolved. Thus, it is still an urgent issue to explore the underlying detailed mechanisms of anxiety disorders and seek potential target for treatment.

It has been demonstrated that physical exercise improves anxiety and depressive‐like behavior effectively through a variety of molecular mechanisms.^[^ ^4^, ^5^ ^]^ For example, activation of cannabinoid receptors mediates the acute anxiolytic and analgesic effects of physical exercise.^[^ ^6^ ^]^ Recent studies have shown that hepatic metabolites after exercise improve synaptic activity in prefrontal cortex by enhancing RNA methylation of synapse‐related transcripts in the brain, thereby preventing anxiety‐like phenotypes.^[^ ^7^ ^]^ The role of RNA m6A has been demonstrated in exercise‐mediated anxiolytic effects. Several kinds of protein posttranslational modifications such as palmitoylation,^[^ ^8^ ^]^ ubiquitination,^[^ ^9^ ^]^ and glycosylation have been reported to play important roles in the development of anxiety and/or depression.^[^ ^10^ ^]^ In addition, dysregulation of S‐nitrosylation of protein post‐translational modification in the central nervous system has been recognized to be associated with brain diseases, including Parkinson's disease,^[^ ^11^ ^]^ Alzheimer's disease,^[^ ^12^ ^]^ and cocaine sensitization.^[^ ^13^ ^]^ However, it is not yet known whether this protein S‐nitrosylation plays a role in the exercise‐induced anxiolytic effects.

It is well accepted that the amygdala is the main nucleus associated with anxiety disorders,^[^ ^14^, ^15^ ^]^ in which the basolateral amygdala (BLA) is recognized as the center for regulating negative emotions. Excessive activation of the BLA projection neurons has been considered to be an important factor leading to anxiety disorders. The activity of pyramidal neurons in the BLA can be regulated by local inhibitory neurons.^[^ ^16^ ^]^ The rapid synaptic inhibition is mediated by type A γ‐aminobutyric acid receptors (GABA_A_Rs) and glycine receptors. Our previous study and the work by other groups have reported that diazepam or deep brain stimulation produces its anxiolytic effect through targeting GABA_A_Rs and its related inhibitory transmission in the BLA,^[^ ^8^, ^17^, ^18^ ^]^ prompting that modulating the surface stability of GABA_A_R on the primary output neurons of BLA may be beneficial to relieve anxiety.

Gephyrin, as a scaffold protein, is the core component of many inhibitory synapses.^[^ ^19^ ^]^ In neurons, gephyrin self‐assembles into a protein scaffold that interacts with the cytoskeleton, which is crucial for anchoring postsynaptic GABA_A_Rs and glycine receptors.^[^ ^20^ ^]^ Genetic knockout of gephyrin disrupts the aggregation of GABA_A_R clusters.^[^ ^21^ ^]^ Gephyrin undergoes a variety of posttranslational modifications that regulate its clustering at inhibitory synapse, including S‐nitrosylation,^[^ ^22^ ^]^ palmitoylation,^[^ ^23^ ^]^ SUMOylation and acetylation.^[^ ^24^ ^]^ S‐nitrosylation refers to the covalent addition of nitric oxide moiety to thiol groups (‐SH).^[^ ^25^ ^]^ In mammalian cells, L‐arginine‐dependent nitric oxide synthases, including neuronal nitric oxide synthase (nNOS), inducible nitric oxide synthase (iNOS), and endothelial nitric oxide synthase (eNOS), are the main sources of endogenous nitric oxide (NO). Abnormal increase in the level of NO triggers nitrosative stress. Previous studies have shown that activation of nNOS‐expressing neurons in the ventromedial prefrontal cortex converts pain signals into anxiety signals through S‐nitrosylation‐induced trafficking of α‐amino‐3‐hydroxy‐5‐methyl‐4‐isoxazole‐propionic acid receptor.^[^ ^26^ ^]^ Considering that posttranslational modification of gephyrin has been implicated in regulating GABA_A_R‐mediated neurotransmission and the dysfunction of GABAergic synaptic transmission is closely associated with anxiety, we asked whether the gephyrin S‐nitrosylation (SNO‐gephyrin) played a role in the pathophysiology of anxiety‐like behaviors.

In the present study, by using electrophysiological, pharmacological, behavioral and viral gene transfer techniques, we identified the association of nNOS‐catalyzed S‐nitrosylation of gephyrin in the BLA with the anxiolytic effect induced by physical exercise. The inhibition of SNO‐gephyrin at Cys212 and Cys284 could promote the GABA_A_R‐mediated synaptic transmission, which in turn produced anxiolytic effects.

## 2 Results

### 2.1 Wheel‐Running Exercise Produces Anxiolytic‐Like Effects via Inhibiting SNO‐Gephyrin

**Figure 1:**
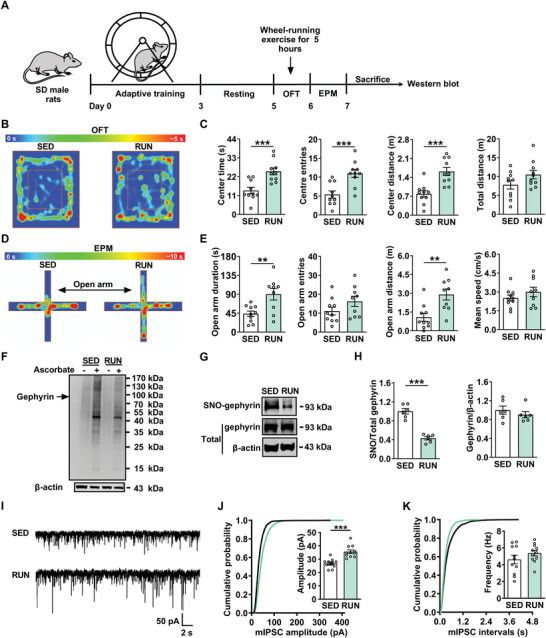
Physical exercise induces anxiolytic‐like effects by downregulation of SNO‐gephyrin. A) Timeline of experimental procedure. B) Representative heatmap from SED and RUN rats in OFT. C) Anxiolytic effects of physical exercise in OFT, including increased time, entries, and distance in the center zone, but not affected locomotor activity (*n* = 10 per group, Student's *t‐*test). D) Representative heatmap from SED and RUN rats in the EPM test. E) Anxiolytic effects of physical exercise in EPM test, including increased duration and distance in the open arms, but not affected locomotor activity (*n* = 9–10 per group, Student's *t‐*test). F) Total level of protein S‐nitrosylation in the BLA of SED and RUN rats. Representative western blot of total level of protein S‐nitrosylation from the BLA of paired SED rats and RUN rats are shown. Ascorbate ‐, without giving reducing buffer ascorbate to reduce the nitrosothiols (the negative control of biotin‐switch method); Ascorbate +, the reducing buffer ascorbate to reduce the nitrosothiols. G) Representative western blot of SNO‐gephyrin from the BLA of paired SED rats and RUN rats are shown. H) The level of SNO‐gephyrin in RUN rats was significantly decreased, but not total expression of gephyrin protein in the BLA (*n* = 6 per group, Student's *t‐*test). I) Representative traces of mIPSCs in the BLA from SED and RUN groups. Scale bar, 2 s, 50 pA. J,K) Physical exercise increased miniature inhibitory postsynaptic currents (mIPSCs) amplitude in RUN rats J), but without change in frequency K) (*n* = 11 cells from 4 rats per group, Student's *t‐*test). All data are presented as mean ± SEM, **P < 0.01, ***P < 0.001. The statistical details can be found in Table S1, Supporting Information.

To investigate whether physical exercise could produce anxiolytic effects, we employed the voluntary wheel‐running exercise as described in previous study.^[^ ^6^ ^]^ First, male rats were fitted with wheel equipment and trained for 3 days. After an adaptation for 2 days with blocked running wheels, rats were then divided into sedentary group (SED) and running group (RUN) based on the distance they ran once the wheels were no longer blocked. Before the behavioral test, the runners underwent a brief five‐hour voluntary wheel‐running (**Figure** **1**A). The figure shows that the wheel‐running exercise produced anxiolytic effects in open field test (OFT) and elevated plus maze (EPM) test. In the OFT, the rats in RUN group spent much more time, entries, and distance in the center zone, with no alteration in locomotor activity (Figure 1B,C). A similar anxiolytic effect of physical exercise was observed in EPM test, including increased time and distance in open arm for RUN rats, without impairment of locomotor ability (Figure 1D,E). The result from western blot showed that physical exercise inhibited total level of protein S‐nitrosylation (Figure 1F). Furthermore, quantitative analysis confirmed that physical exercise decreased the level of SNO‐gephyrin, but not total expression of gephyrin protein compared with that of SED rats (Figure 1G,H). Then, we explored whether physical exercise could enhance the inhibitory synaptic transmission in the BLA. As shown in Figure 1I–K, the amplitude of miniature inhibitory postsynaptic currents (mIPSCs) was increased in RUN group, with no effect on the frequency. These results suggest that physical exercise improves anxiety‐like behaviors in rats by increasing GABAergic neurotransmission in a post‐synaptic manner. More importantly, physical exercise also reduced S‐nitrosylation of gephyrin in the BLA.

### 2.2 S‐Nitrosylated Gephyrin is Elevated in High‐Anxiety Male Rats

**Figure 2:**
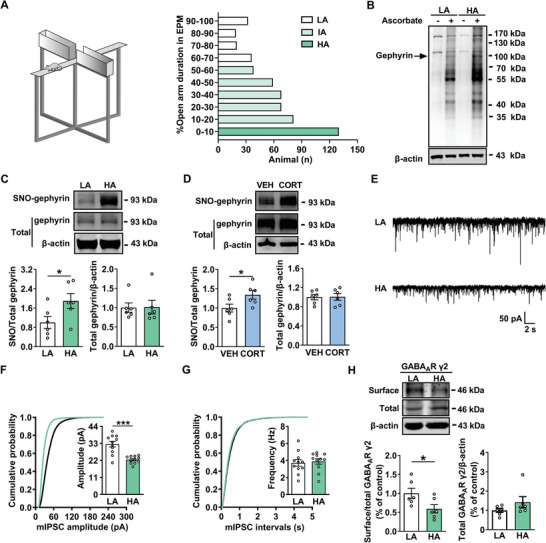
SNO‐gephyrin is higher in high‐anxiety rats. A) The distribution of the time spent in HA (*n* = 130), IA (*n* = 314), and LA (*n* = 107) rats during the EPM test. B) Total level of protein S‐nitrosylation in the BLA of LA and HA rats. Representative western blot of total level of protein S‐nitrosylation from the BLA of paired LA rats and HA rats are shown. C) The expression of gephyrin S‐nitrosylation was increased in the BLA form HA rats (*n* = 6 per group, Student's *t‐*test). D) The S‐nitrosylation of gephyrin in the BLA of rats exposed to corticosterone (CORT) was increased (*n* = 6 per group, Student's *t‐*test). E) Representative traces of mIPSCs in the BLA from LA and HA groups. Scale bar, 2 s, 50 pA. F,G) The amplitude of mIPSCs in HA rats decreased F), but without change in the frequency G) (*n* = 11 cells from 4 rats per group, Student's *t‐*test). H) Western blot results showing decreased ratio of surface GABA_A_R γ2 (sGABA_A_R γ2)/ total GABA_A_R γ2 (tGABA_A_R γ2) in the BLA from HA rats, without alterations in tGABA_A_R γ2 expression (*n* = 6 per group, Student's *t‐*test). All data are presented as the mean ± SEM, *P < 0.05, ***P < 0.001. The statistical details can be found in Table S1 (Supporting Information).

To identify the role of S‐nitrosylation in anxiety‐like behavior, male rats were divided into three groups according to the duration in open arm in EPM test: low‐anxiety (LA) group, intermediate‐anxiety (IA) group and high‐anxiety (HA) group (**Figure** **2**A; Figure S1A–D, Supporting Information). We found that the level of total level of protein S‐nitrosylation was increased in the BLA of HA rats (Figure 2B). To investigate the role of S‐nitrosylation of gephyrin in anxiety‐like behaviors, the level of SNO‐gephyrin was observed in the BLA of HA rats. It was found that there was an increase in the level of SNO‐gephyrin rather than total gephyrin compared with that of LA rats (Figure 2C). In addition, we observed that chronic exposure to corticosterone increased S‐nitrosylation in the BLA of rats, accompanied with anxiety‐like behavior (Figure S2A–I, Supporting Information). SNO‐gephyrin was also increased in corticosterone‐treated rats compared with that in vehicle (VEH) group (Figure 2D). Taken together, these results suggest that SNO‐gephyrin is elevated in high‐anxiety rats.

Since anxiety‐like behavior is closely related to the dysfunction of inhibitory synaptic transmission, we then performed electrophysiological experiments for mIPSCs recordings in the BLA neurons, followed by the measurement of surface expression of GABA_A_R subunits. It was found that the amplitude of mIPSCs was decreased in the BLA neurons of HA rats, without alterations in the mIPSCs frequency (Figure 2E–G). Considering that downregulation of the S‐nitrosylation of gephyrin protein increases the surface expression of GABA_A_R subunits α1, β2/3 and γ2,^[^ ^22^ ^]^ we examined the changes in the expression of GABA_A_R subunits in HA rats. It was shown that surface expression of GABA_A_R γ2 was decreased in the BLA of HA rats compared with that of LA rats (Figure 2H), which was consistent with previous study that γ2 subunit is critical for postsynaptic clustering of GABA_A_Rs.^[^ ^27^ ^]^ No changes were found in the levels of α1, α2, α3 and β2 subunits in HA rats (Figure S3A–D, Supporting Information). Taken together, the experiments above demonstrate that there is an increase in SNO‐gephyrin and a defect in GABAergic transmission in the BLA of HA rats.

### 2.3 S‐Nitrosylation in the BLA Mediates Anxiety‐Like Behavior in Male Rats

**Figure 3:**
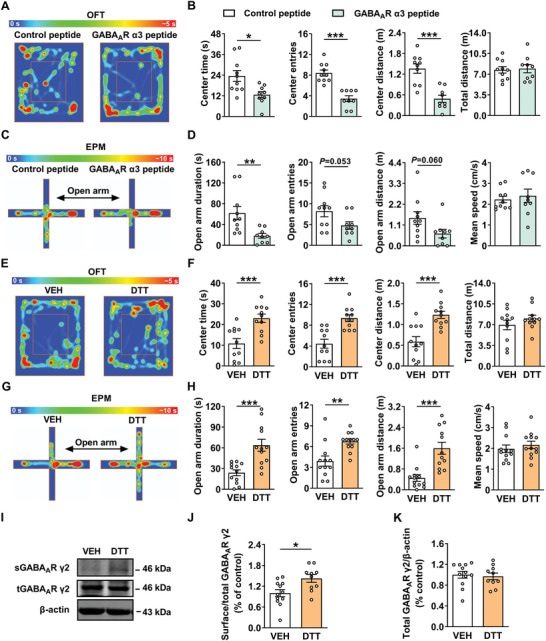
Reducing S‐nitrosylation of protein in the BLA alleviates anxiety‐like behaviors in rats. A) Representative heatmap from control peptide and GABA_A_R α3 peptide treatment rats in OFT. B) GABA_A_R α3 peptide treated rats displayed anxiety‐like behaviors in OFT (*n* = 9–10 per group, Student's *t‐*test). C) Representative heatmap from control peptide and GABA_A_R α3 peptide treatment rats in EPM test. D) GABA_A_R α3 peptide treated rats displayed anxiety‐like behaviors in EPM test (*n* = 9–10 per group, Student's *t‐*test). E) Representative heatmap from vehicle (VEH) and sulfhydryl‐reducing reagents dithiothreitol (DTT) (1.0 mM, 1 µL per side)‐treated rats in OFT. F) DTT‐treatment showed anxiolytic effects in OFT (*n* = 11 per group, Student's *t‐*test). G) Representative heatmap from VEH and DTT‐treated rats in EPM test. H) DTT‐treatment showed anxiolytic effects in EPM test (*n* = 12 per group, Student's *t‐*test). I) Representative western blot of sGABA_A_R γ2, tGABA_A_R γ2 protein with VEH and DTT treatment in the BLA. J, K) Western blot results showing DTT increased the ratio of sGABA_A_R γ2/tGABA_A_R γ2, but not tGABA_A_R γ2 expression in the BLA (*n* = 10–12 per group, Student's *t‐*test). All data are presented as mean ± SEM, *P < 0.05, **P < 0.01, ***P < 0.001. The statistical details can be found in Table S1 (Supporting Information).

It has been demonstrated that the interaction between gephyrin and GABA_A_R is crucial for the clustering of GABA_A_R.^[^ ^28^ ^]^ Next, GABA_A_R α3 peptide, a GABA_A_R α3 subunit‐derived peptide with high affinity for gephyrin, was used to disrupt the interaction between gephyrin and GABA_A_R.^[^ ^8^ ^]^ It was found that microinjection of GABA_A_R α3 peptide (100 µM, 1 µL per side) into the BLA reduced the center exploration of rats in OFT, including decreases in time, entries and distance in the center zone compared with that of control peptide (**Figure** **3**A,B). A similar result was observed in EPM test, including reductions of open arm duration, open arm entries, and open arm distance of HA rats (Figure 3C,D). In conclusion, interrupting gephyrin‐GABA_A_Rs interaction caused increased anxiety levels in rats.

Previous studies have shown that GABA_A_R α3 peptide aggravates anxiety‐like behaviors in rats by impairing GABAergic transmission, while downregulation of SNO‐gephyrin increases the number of GABA_A_R cluster.^[^ ^8^, ^22^ ^]^ To determine whether the reduction of S‐nitrosylation could produce anxiolytic effect and reverse the decreased surface expression of GABA_A_R γ2, sulfhydryl‐reducing reagents dithiothreitol (DTT) was used to inhibit S‐nitrosylation of proteins in the BLA of rats. We found that microinjection of DTT (1.0 mM, 1 µL per side) into the BLA reduced the level of SNO‐gephyrin (Figure S4A,B, Supporting Information), together with the improvement of anxiety‐like behaviors compared with that of vehicle group, which was demonstrated by their increased center time, center entries and center distance in the center zone of OFT, without changes in locomotor activity (Figure 3E,F). Similar results were observed in DTT‐treated rats in EPM test (Figure 3G,H). In addition, the surface level of GABA_A_R γ2 was elevated by downregulating SNO‐gephyrin with DTT, without alteration in total expression of GABA_A_R γ2 (Figure 3I–K). These results suggest that the inhibition of protein S‐nitrosylation in the BLA alleviates anxiety‐like behaviors and rescues the sGABA_A_R γ2 expression deficit in rats.

Considering that DTT is a potent reducing agent, it may produce off‐target effects that induce neuronal apoptosis in the BLA and produce anxiolytic effects in rats. We detected whether there was any change in the level of apoptotic factors in the BLA after DTT treatment. The results showed that, compared with the vehicle group, microinjection of DTT into the BLA did not alter the protein expression of a variety of apoptotic factors, including caspase 3, cleaved caspase 3, and B Cellular lymphoma 2 (Bcl‐2) (Figure S4C–F, Supporting Information). Correspondingly, microinjection of DTT into the BLA did not cause changes in the protein level of necroptosis factor receptor interacting protein 3 (RIP3) and pyroptosis factor caspase 1 (Figure S4E–H, Supporting Information). These results exclude the possibility that the anxiolytic effects induced by DTT treatment in the dose above are resulted from neuronal apoptosis in the BLA of rats.

Taken together, these observations indicate that enhanced gephyrin nitrosylation is an important cause of anxiety, and pharmacological inhibition on gephyrin nitrosylation can increase the surface expression of GABA_A_R and alleviate anxiety‐like behaviors in rats.

### 2.4 Inhibition of Protein S‐Nitrosylation of Gephyrin at Cys212 or Cys284 Alleviates Anxiety‐Like Behaviors in Male Rats

**Figure 4:**
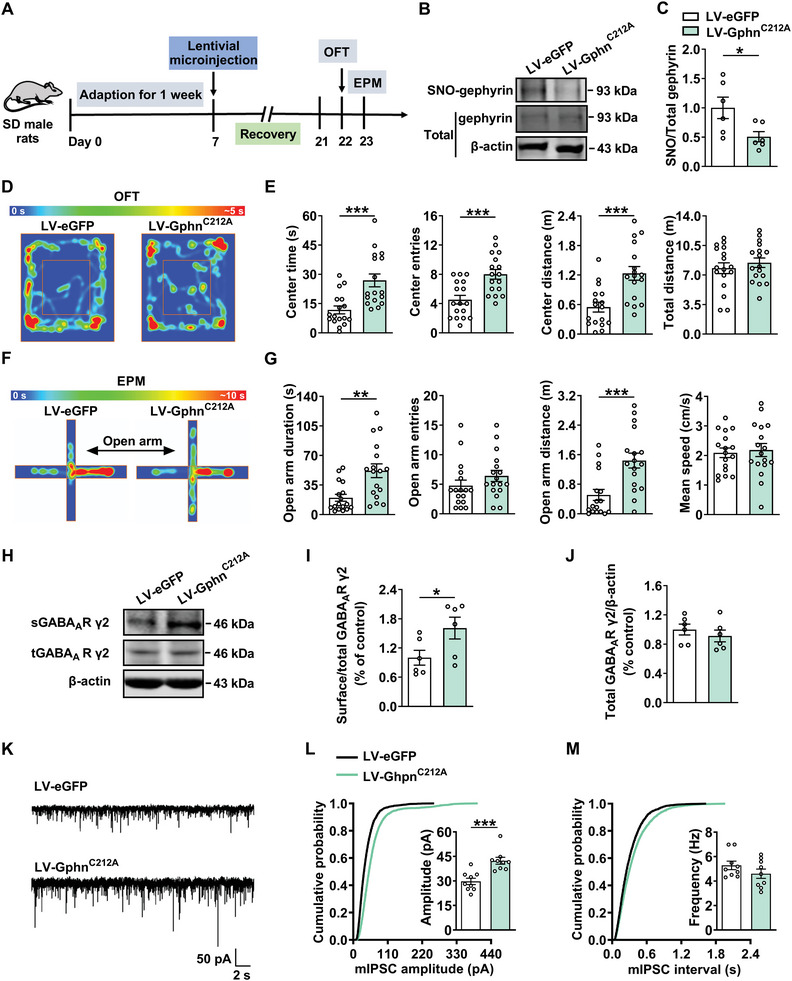
Inhibition of gephyrin S‐nitrosylation by Cys212 mutation alleviates anxiety‐like behaviors. A) Experimental timeline for lentivirus injection and behavioral tests. B) Representative western blot of SNO‐gephyrin and total gephyrin protein from LV‐eGFP and LV‐Gphn^C212A^ groups. C) Western blot results showing that mutation Cys212 decreased SNO‐gephyrin (*n* = 6 per group, Student's *t‐*test). D,F) Representative heatmap from LV‐eGFP and LV‐ Gphn^C212A^ rats in OFT D) and EPM test F). E,G) Mutation of the SNO‐gephyrin locus, Cys212, alleviated anxiety‐like behaviors in rats in OFT (E) and EPM test (G) (*n* = 17 per group, Student's *t‐*test). H) Representative western blot of sGABA_A_R γ2, tGABA_A_R γ2 in the BLA of rats injected with LV‐Gphn^C212A^. I,J) Western blot results showing that mutation of Cys212 at SNO‐gephyrin increased the ratio of sGABA_A_R γ2/tGABA_A_R γ2, but not tGABA_A_R γ2 expression in the BLA (*n* = 6 rats per group, Student's *t‐*test). K) Representative traces of mIPSCs in the BLA from LV‐eGFP and LV‐Gphn^C212A^ groups. Scale bar, 2 s, 50 pA. L,M) The amplitude of mIPSCs in LV‐Gphn^C212A^ group was increased L), but without change in the frequency M) (*n* = 9 cells from 4–6 rats per group, Student's *t‐*test). All data are presented as mean ± SEM, *P < 0.05, **P < 0.01, ***P < 0.001. The statistical details can be found in Table S1 (Supporting Information).

Next, we asked whether S‐nitrosylation of gephyrin at specific cysteine residues was involved in anxiety‐like behaviors. Since the C‐terminal in gephyrin is considered to be closely associated with various post‐translational modifications, we focused on the functional cysteine residues at C‐terminal,^[^ ^23^, ^29^ ^]^ such as Cys212 and Cys284, which are the essential residues for S‐nitrosylation of gephyrin. We constructed a lentivirus encoding a gephyrin mutant, in which Cys212 was replaced by alanine (LV‐Gphn^C212A^), and successfully expressed in the BLA of rats (**Figure** **4**A). Western blot analysis showed that LV‐Gphn^C212A^ increased gephyrin expression compared with that of LV‐eGFP group (Figure S5A,B, Supporting Information). After two weeks of microinjection of LV‐ Gphn^C212A^ into the BLA of male rats, the S‐nitrosylation of gephyrin was reduced in the BLA of rats compared with that of LV‐eGFP group (Figure 4B,C; Figure S5C, Supporting Information), demonstrating that Cys212 is one of the main sites for S‐nitrosylation of gephyrin. Then, the effect of Cys212 mutation on anxiety‐like behaviors was evaluated. As shown in Figure 4D,E, LV‐Gphn^C212A^ increased the time, entries, and distance in the central area in OFT compared with that in LV‐eGFP group. In the EPM test, the time and distance in the open arm were also increased in LV‐Gphn^C212A^ group, without change the entries entering in open arm (Figure 4F,G). Furthermore, we found that surface expression of GABA_A_R γ2 was elevated in the rats of LV‐Gphn^C212A^ group (Figure 4H–J). Considering that LV‐Gphn^C212A^ increased the surface level of GABA_A_R γ2 subunit, we asked whether the inhibitory synaptic transmission was affected. It was shown that the amplitude of mIPSCs was increased in the LV‐Gphn^C212A^ group compared with that of LV‐eGFP group, with no change in the frequency (Figure 4K–M), indicating that sGABA_A_R γ2 expression modulated by inhibition of SNO‐gephyrin at Cys212 is essential for improving anxiety‐like behaviors.

Similar lentivirus encoding a gephyrin mutant in which Cys284 was replaced by alanine (LV‐Gphn^C284A^) was constructed to determine the role of S‐nitrosylation at Cys284 in the functional domain of gephyrin in modulating anxiety‐like behaviors. Two weeks after injection of LV‐Gphn^C284A^, the expression of gephyrin was increased (Figure S6A,B, Supporting Information), but the S‐nitrosylation of gephyrin was reduced in LV‐Gphn^C284A^ group, suggesting that Cys284 is the other key site for S‐nitrosylation of gephyrin (Figure S6C–E, Supporting Information). Behavioral tests showed that the central exploration in the OFT, including center time, center entries and center distance were increased in LV‐Gphn^C284A^ group compared with that in LV‐eGFP group, although total movement parameters did not differ (Figure S6F,G, Supporting Information). Meanwhile, LV‐Gphn^C284A^‐treated rats displayed increased exploration in the open arm in EPM compared with that in LV‐eGFP group (Figure S6H,I, Supporting Information). LV‐Gphn^C284A^ increased the surface expression of GABA_A_R γ2 compared with that in LV‐eGFP group, without effect on the total expression of GABA_A_R γ2 (Figure S6J–L, Supporting Information). Correspondingly, LV‐Gphn^C284A^ increased the mIPSCs amplitude, while the mIPSCs frequency was not changed (Figure S6M–O, Supporting Information). These results indicate that the inhibition of SNO‐gephyrin at Cys212 or Cys284 increased surface level of GABA_A_R γ2, which contributes to the anxiolytic effect.

Available evidence indicates that Cys212 and Cys284, the two nitrosylation sites of gephyrin identified in the present study, may also undergo palmitoylation (Pal‐gephyrin)^[^ ^30^ ^]^ by the catalysis of the enzyme Asp‐His‐His‐Cys (DHHC)−12.^[^ ^23^ ^]^ Studies have shown that nitrosylation at the same cysteine sites inhibits palmitoylation at these sites, and reduced palmitoylation leads to increased nitrosylation.^[^ ^31^ ^]^ We wondered whether mutant nitrosylation sites could change the palmitoylation level of gephyrin. The results showed that after microinjection of LV‐Gphn^C212A^ or LV‐Gphn^C284A^ into the BLA, gephyrin palmitoylation was not altered significantly in the LV‐Gphn^C212A^ and LV‐Gphn^C284A^ groups compared with the LV‐eGFP group (Figure S7A,B, Supporting Information). The level of total gephyrin was not altered (Figure S7C, Supporting Information). Considering the competition between nitrosylation and palmitoylation of gephyrin at the same sites, we further clarified whether gephyrin palmitoylation was involved in this process by impairing the interaction between gephyrin and GABA_A_R to cause GABA inhibitory signaling defects. Co‐immunoprecipitation was then used to observe whether the interaction between gephyrin and GABA_A_R is changed due to mutating the nitrosylation sites at Cys212 or Cys284 of gephyrin. The results showed that, compared with the LV‐eGFP group, the interaction between gephyrin and GABA_A_R γ2 in the LV‐Gphn^C212A^ and LV‐Gphn^C284A^ groups was not destroyed (Figure S7D,E, Supporting Information). The expression level of total GABA_A_R γ2 was not altered (Figure S7F, Supporting Information). Therefore, we conclude that mutating the gephyrin nitrosylation sites has minor effect on gephyrin palmitoylation.

Taken together, these results suggest that inhibiting S‐nitrosylation of Cys212 or Cys284 in gephyrin enhances inhibitory neurotransmission, which may explain the mechanism by which downregulation of SNO‐gephyrin produces anxiolytic effects.

### 2.5 Inhibition of nNOS Ameliorates Anxiety‐Like Behaviors in Male Rats via Reducing the Expression of SNO‐Gephyrin

**Figure 5:**
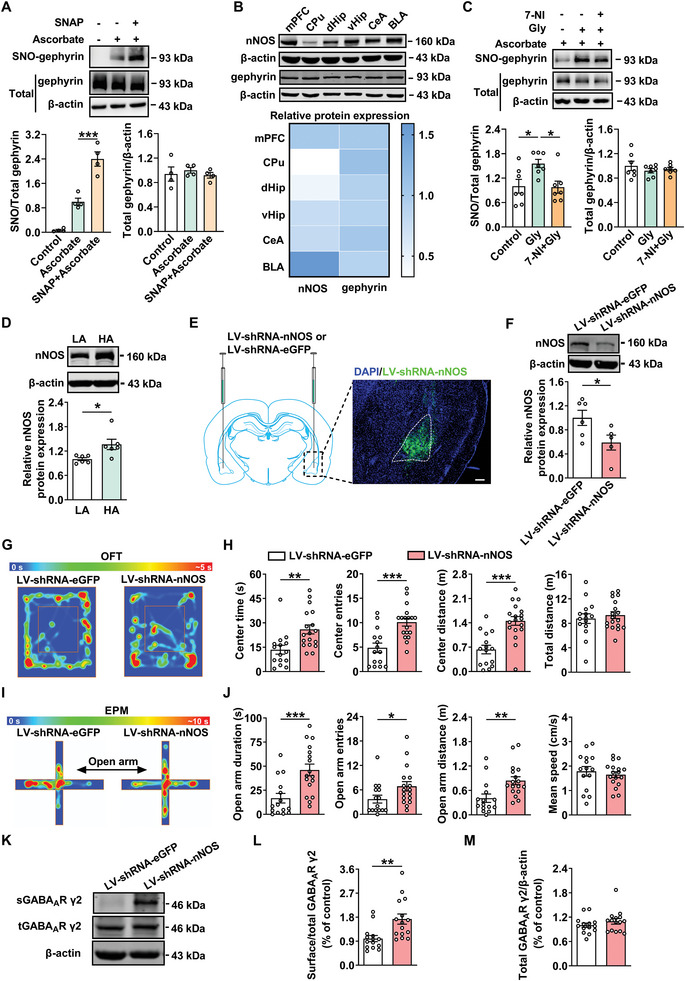
SNO‐gephyrin requires nNOS, downregulation or inhibition of nNOS in the BLA alleviates anxiety in male rats. A) S‐nitrosylation of gephyrin demonstrated by biotin switch method. Ascorbate dependence demonstrates specificity of the signal and increased S‐nitrosylation of gephyrin by in vivo administration of S‐Nitroso‐N‐acetyl‐DL‐penicillamine (SNAP) (100 µM) with the BLA slices, the NO donor (*n* = 4 per group, one‐way ANOVA followed by Bonferroni's post hoc test). B) Representative immunoblots and quantification of gephyrin and nNOS within the medial prefrontal cortex (mPFC), caudate putamen (CPu), ventral hippocampus (vHip), dorsal hippocampus (dHip), central amygdala (CeA) and the BLA (*n* = 6 per group). C) Activation of nNOS by glycine (Gly) (200 µM) treatment with the BLA slices increased the level of gephyrin S‐nitrosylation, while abolished by 7‐nitroindazole (7‐NI) (100 µM), the inhibitor of nNOS (*n* = 7 per group, one‐way ANOVA followed by Bonferroni's post hoc test). D) The protein expression of nNOS in the BLA was significantly increased in HA rats (*n* = 6 per group, Student's *t‐*test). E) Schematic sagittal section of the BLA representing the microinjection site of lentivirus vector. Blue, 4′,6‐diamidino‐2‐phenylindole (DAPI); green, LV‐shRNA‐nNOS. Scale bar: 150 µm. F) Representative western blot of nNOS in the BLA with LV‐shRNA‐eGFP or LV‐shRNA‐nNOS injection. The quantification showing that the LV‐shRNA‐nNOS decreased the expression of nNOS in the BLA (*n* = 5–6 per group, Student's *t‐*test). G,I) Representative heatmap from LV‐shRNA‐eGFP and LV‐shRNA‐nNOS rats in OFT G) and EPM test I). H,J) The rats with LV‐shRNA‐nNOS showed reduced anxiety‐like behaviors compared with LV‐shRNA‐eGFP in OFT H) and EPM test J) (*n* = 15–18 per group, Student's *t‐*test). K) Representative western blot of sGABA_A_R γ2, tGABA_A_R γ2 protein in the BLA with LV‐shRNA‐nNOS injection. L,M) Western blot results showing LV‐shRNA‐nNOS increased the ratio of sGABA_A_R γ2/tGABA_A_R γ2, but not tGABA_A_R γ2 expression in the BLA (*n* = 14–15 per group, Student's *t‐*test). All data are presented as mean ± SEM, *P < 0.05, **P < 0.01, ***P < 0.001. The statistical details can be found in Table S1 (Supporting Information).

The sources of nitrosylation groups may include exogenous or endogenous nitroxides.^[^ ^32^ ^]^ Therefore, we explored the sources that mediate SNO‐gephyrin via treatment BLA slices with S‐Nitroso‐N‐acetyl‐DL‐penicillamine (SNAP), the nitric oxide donor, followed by biotin switch assays on gephyrin. The results showed that SNAP (100 µM) elicited S‐nitrosylation of gephyrin (**Figure** **5**A), and the level of nNOS was highest in the BLA (Figure 5B). To investigate whether endogenous S‐nitrosylation of gephyrin was nNOS‐dependent, the BLA slices were treated with glycine (Gly, 200 µM) in the presence of 7‐Nitroindazole (7‐NI), a selective nNOS inhibitor. The results showed that 7‐NI (100 µM) inhibited SNO‐gephyrin (Figure 5C), suggesting that SNO‐gephyrin is mediated by nNOS. By use of quantitative PCR and western blot analysis, our results showed that the expressions of nNOS mRNA and protein were much higher in the BLA of HA rats, while the levels of eNOS mRNA and iNOS protein were unchanged compared with that of LA rats (Figure 5D; Figure S8A–C, Supporting Information). Furthermore, physical exercise decreased the expression of nNOS protein in the BLA (Figure S8D, Supporting Information). These results suggest that SNO‐gephyrin is dependent on the level of nNOS in HA rats. Next, we asked whether NO‐cGMP‐PKG signaling was involved in the anxiety‐like behaviors of rats. It was shown that microinjection of PKG inhibitor KT5823 (1 µM, 1 µL per side) into the BLA did not alter the time, entries and distance in the center zone in OFT (Figure S8E,F, Supporting Information), and the duration, entries and distance in the open arm in EPM test (Figure S8G,H, Supporting Information). These results suggest that S‐nitrosylation of gephyrin is dependent on nNOS‐mediated endogenous NO production, which induces anxiety‐like behavior.

To address the contribution of nNOS‐mediated SNO‐gephyrin to anxiety‐like behaviors, lentivirus (LV)‐mediated gene transfer of constructs containing short hairpin RNA targeting nNOS (shRNA‐nNOS) was injected into BLA to reduce nNOS expression (Figure 5E,F; Figure S9A, Supporting Information). As shown in Figure S9B–D (Supporting Information), knockdown of nNOS in the BLA down‐regulated SNO‐gephyrin. The behavioral tests were performed two weeks after virus injection, and showed reductions in anxiety‐like behaviors in LV‐shRNA‐nNOS‐treated rats compared with that in LV‐shRNA‐eGFP group, including increased time, entries and distance in the center zone of OFT (Figure 5G,H) and in the open arm of EPM (Figure 5I,J). Considering that SNO‐gephyrin regulates surface expression of GABA_A_R γ2, we then examined the effect of nNOS‐knockdown on the surface expression of GABA_A_R γ2. It was found that the surface expression of GABA_A_R γ2 was increased in LV‐shRNA‐nNOS compared with that in control LV‐shRNA‐eGFP group, without changes in total level of GABA_A_R γ2 (Figure 5K–M), suggesting that nNOS knockdown‐mediated reduction of SNO‐gephyrin ameliorates anxiety‐like behaviors by increasing surface clustering of GABA_A_R γ2.

To further verify the role of nNOS‐mediated SNO‐gephyrin, 7‐NI, a specific nNOS inhibitor was employed to investigate the effect of nNOS on anxiety‐like behaviors. It was found that 7‐NI (100 µM, 1 µL per side) increased the duration, entries and distance in the center zone of OFT compared with that in vehicle group (Figure S9E,F, Supporting Information). The similar results were observed in EPM test (Figure S9G,H, Supporting Information). However, locomotor activity was not influenced by 7‐NI. The surface expression of GABA_A_R γ2 was upregulated in the BLA of 7‐NI‐treated rats compared with that of vehicle group (Figure S9I–K, Supporting Information). These results suggest that downregulation of nNOS produces anxiolytic effect, which is at least partly due to the increase in GABA_A_Rs clustering. The nitrosylation of gephyrin is dependent on nNOS, and physical exercise suppresses SNO‐gephyrin through reducing nNOS.

### 2.6 Overexpression of nNOS Abolishes the Anxiolytic Effect of Physical Exercise

**Figure 6:**
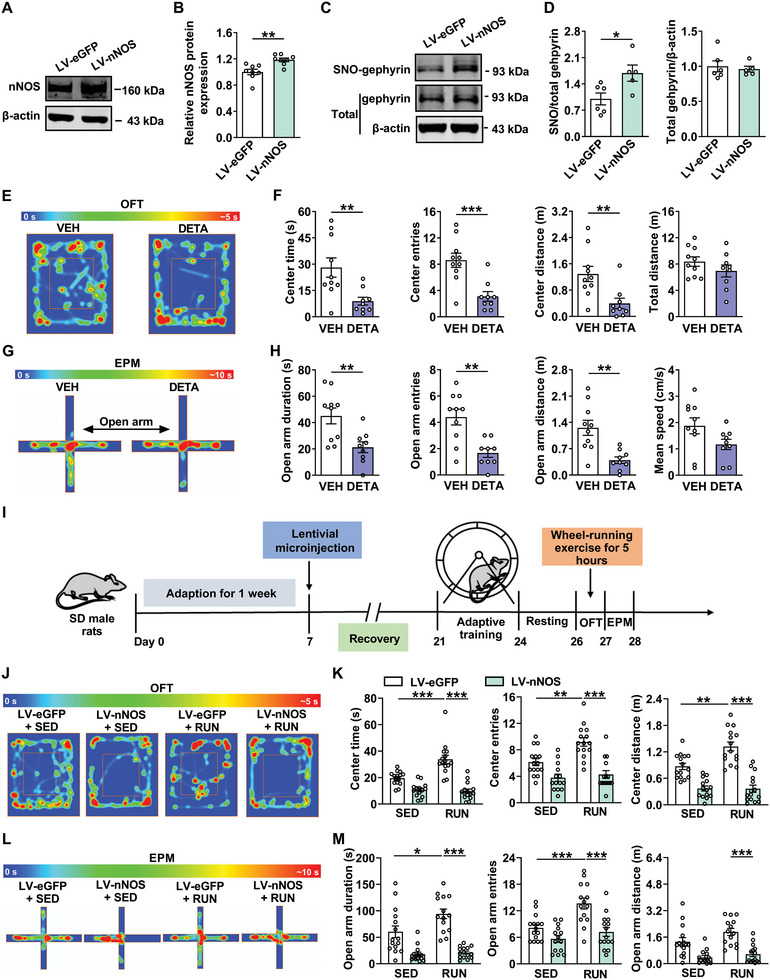
Overexpression of nNOS abolishes anxiolytic effects of physical exercise. A) Representative western blot of nNOS in the BLA with LV‐nNOS injection. B) The quantification showing that nNOS expression was increased in the BLA by LV‐nNOS (*n* = 8 per group, Student's *t‐*test). C) Representative western blot of SNO‐gephyrin and total gephyrin in the BLA with LV‐nNOS injection. D) The quantification showing that LV‐nNOS increased the expression of SNO‐gephyrin in the BLA (*n* = 5–6 per group, Student's *t‐*test). E,G) Representative heatmap from vehicle and DETA‐NONOate (DETA) (100 µM, 1 µL per side)‐treated rats in OFT E) and EPM test G). F,H) Administration of DETA induced anxiety‐like behaviors in OFT F) and EPM test H) (*n* = 9–10 per group, Student's *t‐*test). I) Experimental timeline for lentivirus injection, wheel‐running exercise and behavioral tests. J,L) Representative heatmap in OFT J) and EPM test L) from LV‐eGPF + SED, LV‐nNOS + SED, LV‐eGFP + RUN, and LV‐nNOS + RUN groups. K,M) In the OFT K) and EPM test M), nNOS overexpression abolished the anxiolytic effect produced by wheel‐running exercise (*n* = 14–15 per group, two‐way ANOVA followed by Bonferroni's post hoc test). All data are presented as mean ± SEM, *P < 0.05, **P < 0.01, ***P < 0.001. The statistical details can be found in Table S1 (Supporting Information).

Next, we wondered whether nNOS‐mediated increase in the expression of SNO‐gephyrin would increase anxiety‐like behaviors in rats. The lentiviruses were used to overexpress nNOS in the BLA of rats (Figure S10A,B, Supporting Information; **Figure** **6**A,B), and we found that the level of SNO‐gephyrin was increased by LV‐nNOS (Figure 6C,D). Overexpression of nNOS in the BLA reduced the time, entries and distance in the center zone of OFT compared with LV‐eGFP group (Figure S10C,D, Supporting Information). A similar effect was observed in the EPM test by overexpression of nNOS in the BLA (Figure S10E,F, Supporting Information), suggesting that the overexpression of nNOS is sufficient to induce anxiety‐like behaviors. The up‐regulation of nNOS decreased the surface expression of GABA_A_R γ2 (Figure S10G–I, Supporting Information), which is consistent with the decrease in surface expression of GABA_A_R γ2 in HA rats. Next, DETA‐NONOate (DETA), a NO donor was microinjected into the BLA. Treatment with DETA (100 µM) reduced the duration, entries and distance in the center zone of OFT (Figure 6E,F). The result of EPM test was consistent with that in OFT (Figure 6G,H). In addition, DETA reduced the surface expression of GABA_A_R γ2 in the BLA (Figure S10J–L, Supporting Information). Together, the findings indicated that SNO‐gephyrin aggravates anxiety‐like behaviors by reducing the surface expression of GABA_A_R γ2.

Next, we explored whether overexpression of nNOS abolished the anxiolytic effect of physical exercise. Our results showed that physical exercise produced anxiolytic effect in LV‐eGFP + RUN group compared with that in LV‐eGFP + SED group, while overexpression of nNOS abolished the anxiolytic effect induced by physical exercise in OFT (Figure 6I–K) and EPM test (Figure 6L,M), without influence on locomotor activity (Figure S10M, Supporting Information). Treatment with LV‐nNOS reduced the mean speed of movement in EPM of rats (Figure S10N, Supporting Information). These data suggest that the anxiolytic effects of physical exercise are mediated by reducing the level of SNO‐gephyrin in the BLA.

## 3 Discussion

**Figure 7:**
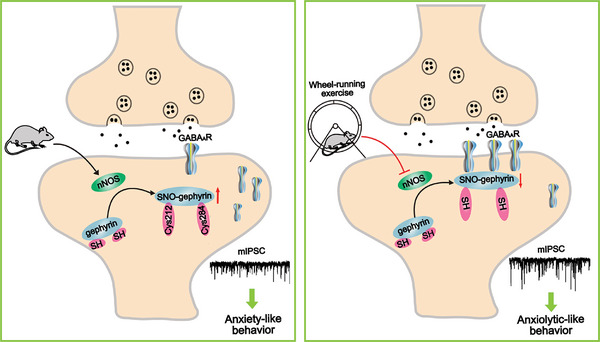
Voluntary wheel‐running exercise exerts anxiolytic effects through downregulation of SNO‐gephyrin. SNO‐gephyrin reduces the surface expression of GABA_A_Rs and impairs GABA_A_R‐dependent inhibitory neurotransmission in the BLA, resulting in anxiety disorders, which has been alleviated by wheel‐running exercise.

Our study identified an essential role of S‐nitrosylation of gephyrin in anxiety‐like behaviors and demonstrated that the regulation of this S‐nitrosylation mediated the mechanism underlying the anxiolytic effect induced by physical exercise (**Figure** **7**). The site‐specific mutation decreased the level of SNO‐gephyrin, and increased the surface expression of GABA_A_R γ2 and inhibitory neurotransmission, resulting in anxiolytic‐like behaviors. In addition, the anxiolytic effect of physical exercise was prevented by overexpression of nNOS. Either pharmacological inhibition or genetic knockdown of nNOS decreased SNO‐gephyrin and alleviated the anxiety‐like behaviors of rats. Our study found a previously unrecognized role of protein nitrosylation in physical exercise‐induced anxiolytic effect.

Physical exercise has been widely proven to produce anxiolytic effect.^[^ ^33^ ^]^ A meta‐analysis shows that physical exercise is effective in improving anxiety symptoms in people with stress‐related disorders.^[^ ^34^ ^]^ The beneficial effects of exercise are related to the release of neurotransmitters, extracellular vesicles and neurotrophic factors. For example, regular aerobic exercise increases serotonin release and enhance long‐term potentiation in the anterior cingulate cortex, ultimately improving pain and concomitant anxiety behavior via activation of 5‐HT1A and 5‐HT7 receptors.^[^ ^35^ ^]^ Voluntary wheel‐running exercise increases the level of dopamine in the striatum of mice,^[^ ^36^ ^]^ and the selenium transport to promote hippocampal neurogenesis, thereby reversing cognitive impairment caused by aging.^[^ ^37^ ^]^ In addition, exercise induces the release of extracellular vesicles into circulation, upregulates miRNA profiles associated with neurotrophic signaling, and improves restraint stress‐induced anxiety‐like behaviors in mice.^[^ ^38^ ^]^ Relatively short‐term running exercise increases the expression of peroxisome proliferator‐activated receptor‐γ coactivator‐1α and brain‐derived neurotrophic factor in the ventral hippocampus and relieves anxiety.^[^ ^39^ ^]^ Emerging evidence has pointed out that epigenetic regulation of RNA N(6)‐methyladenosine (m6A) is involved in the anxiolytic effects of physical exercise.^[^ ^7^, ^40^ ^]^ Specifically, exercise‐induced brain RNA methylation inhibits the expression of fragile X mental retardation protein, thereby activating the mTOR pathway to maintain cortical neural activity and axon myelination, which contributes to the increase in the stress resilience in adolescent mice.^[^ ^40^ ^]^ In the present study, we demonstrated that the level of S‐nitrosylation was increased in HA rats, and a downregulation of SNO‐gephyrin was associated with the anxiolytic effect of voluntary wheel‐running exercise. Furthermore, we identified that the specific sites of S‐nitrosylation in gephyrin was related to anxious state. Thus, our work elucidated that the post‐translational modification of S‐nitrosylation of proteins contributed to the beneficial effects of physical exercise, which extends current neurobiological mechanism underlying the anxiolytic action of physical exercise. Physical exercise can improve physical performance, mental state, overall health status and happiness. There is currently no consensus on the ideal frequency, intensity, type, and duration of exercise for the treatment of anxiety disorder, and future research should address these issues by directly assessing the effects of different types of exercise regimens on anxiety disorder.

S‐nitrosylation has been considered to regulate the function of proteins, which may be involved in the pathology of a variety of diseases. In the central nervous system, protein S‐nitrosylation has been reported to be associated with neurodegenerative diseases, such as Parkinson's disease and Alzheimer's disease. For example, previous studies have shown that S‐nitrosylation of cyclin‐dependent kinase 5 inhibits neurotoxicity and autophagy in a mouse model of Parkinson's disease.^[^ ^41^ ^]^ Increased S‐nitrosylation of mitochondrial complex I by nitrite is protective against Parkinson's disease.^[^ ^42^ ^]^ Aberrant transnitrosylation triggers excessive mitochondrial fragmentation in Alzheimer's disease.^[^ ^43^, ^44^ ^]^ Our present study suggests that protein S‐nitrosylation is involved in the generation of anxiety‐like behaviors. S‐nitrosylation of gephyrin in the BLA of HA rats was up‐regulated, which could be downregulated by physical exercise. S‐nitrosylation of gephyrin has been demonstrated to regulate GABA_A_R trafficking.^[^ ^22^ ^]^ Our previous study has found that the increase in GABA_A_R trafficking to cell membrane rapidly improves the anxiety‐like behaviors of rats.^[^ ^45^ ^]^ In the present study, we found the deficits in surface expression of GABA_A_R and GABAergic transmission in the BLA of HA rats. These results support the notion that increased S‐nitrosylation of gephyrin attenuates the surface expression of GABA_A_R, which leads to impaired GABAergic transmission, and ultimately resulting in the anxiety‐like behaviors.

In the present study, SNO‐gephyrin was significantly increased in the BLA of HA rats, and most of the nitrosylated gephyrin remained in the cytoplasm. We first microinjected the nNOS inhibitor into the BLA and produced a significant anxiolytic effect. This effect depends on the down‐regulation of SNO‐gephyrin, but it cannot be ruled out that the down‐regulation of SNO‐gephyrin causes an increase in gephyrin palmitoylation. These two processes together cause increased membrane expression of GABA_A_R γ2, thereby producing anxiolytic behavioral effects. Next, when site mutation viruses were used to reduce SNO‐gephyrin, the interaction between gephyrin and GABA_A_R may be enhanced, thereby increasing the clustering of GABA_A_R at inhibitory synapses. As a result, GABAergic neurotransmission deficit has been improved, to produce anxiolytic effect. Since in our co‐immunoprecipitation results, the binding between gephyrin and GABA_A_R γ2 tended to be enhanced after mutating the nitrosylation site of gephyrin, we could not exclude the potential role of other proteins which can also bind to GABA_A_R and regulate GABA_A_R clustering. For example, postsynaptic anchoring of GABA_A_Rs is ensured by complex interactions between the scaffolding proteins gephyrin, neuroligin‐2, and collybistin.^[^ ^46^ ^]^ Direct interactions between these proteins and GABA_A_R subunits contribute to the synapse‐specific distribution of GABA_A_R.^[^ ^46^ ^]^ Targeted deletion of neuroligin‐2 disrupts the perisynaptic accumulation of postsynaptic GABA_A_R and its anchoring protein gephyrin. Gephyrin, GABA_A_R‐related protein (GABA_A_RAP) and collybistin can directly interact with GABA_A_R to regulate GABA_A_R clustering.^[^ ^47^, ^48^ ^]^ Changes in the nitrosylation of gephyrin may also indirectly cause changes in GABA_A_R γ2 membrane expression by affecting its binding strength to GABA_A_RAP, collybistin and neuroligin‐2, thereby regulating anxiety‐like behaviors. Thus, more evidence needs to be provided in future studies to explain the regulation of anxiety by SNO‐gephyrin.

The present study also provided evidence for an important role of gephyrin S‐nitrosylation in the stabilization of surface GABA_A_Rs in BLA. The disulfide bond reducing agent DTT was used to maintain the reduced state of ‐SH in the protein. This treatment attenuated the S‐nitrosylation of gephyrin and increased the surface expression of GABA_A_R, which contributed to the anxiolytic effect. In addition, blockade of gephyrin‐GABA_A_R signaling using GABA_A_R α3 subunit‐derived peptides aggravated anxiety‐like behaviors, which was in consistence with our previous findings. Several novel denitrosylases have been identified, including glutaredoxin,^[^ ^49^ ^]^ thioredoxin‐related protein of 14 kDa,^[^ ^50^ ^]^ and thioredoxin.^[^ ^51^ ^]^ However, due to the limited use of these enzymes in vivo, we did not perform in vivo experiments to explore whether these endogenous enzymes could abolish the effect of SNO‐gephyrin on GABA_A_R trafficking, GABAergic transmission and anxiety‐like behaviors. Further evaluation is required to examine the effect of denitrosases on anxiety‐like behaviors.

The cysteine residues targeted by S‐nitrosylation conform to the acid‐base consensus motif, but the acid‐base motif derived from the tertiary structure also induces protein S‐nitrosylation.^[^ ^32^, ^52^, ^53^ ^]^ Tertiary protein structure and/or protein‐protein interactions may affect the relative hydrophobicity of regions surrounding target thiols and regulate protein S‐nitrosylation.^[^ ^53^ ^]^ In the present study, we identified the S‐nitrosylated residues of gephyrin that involved in anxiety‐like behaviors, and confirmed that the precise targeting sites for this S‐nitrosylation were Cys212 and Cys284. We further demonstrated that both Gphn^C212A^ and Gphn^C284A^ mutants produced anxiolytic effects. However, there are three cysteine residues in the functional C‐terminal of gephyrin, Cys212, Cys284 and Cys293,^[^ ^23^ ^]^ we could not excluded the role of S‐nitrosylation at Cys293 of gephyrin in anxiety disorders.

Gephyrin consists of three domains, namely the conserved N‐terminal G domain and the C‐terminal E domain, connected by a central (C) domain.^[^ ^54^ ^]^ The functional complexity and diversity of gephyrin is regulated by alternative splicing in a tissue‐specific manner. Eleven alternatively spliced exons were found in rat, mouse, and human, and most of the resulting mRNAs expressed full‐length gephyrin, spliced gephyrin with C2 and C6 cassette or an additional C4 exon in the C domain variants are the most common splice variants. In rat, the gephyrin C domain contains C3, C4, and C5 cassette.^[^ ^55^ ^]^ Mass spectrometry analysis shows that splicing variants containing C2, C6, and C4 cassette are mainly expressed in the rat brain, and splicing variants containing C3, C4, and C5 cassette were also expressed.^[^ ^56^, ^57^ ^]^ However, it is not yet clear which splicing variant is the most abundant in the BLA of rat. Through querying the Uniprot and GenBank databases, we found 23 gephyrin isomers in rats, among which the 212th amino acid of gephyrin X1, X4, X5, X6, X7, X9, X13, and X15 isomers is cysteine. The isomers of gephyrin X14, X16, X17, X19, and X21 have amino acids at positions 212 and 284, all of which are cysteine. These findings indicate that the majority of gephyrin isomers in rats have cysteine as the amino acid at site 212.

Gephyrin can undergo both S‐nitrosylation and S‐palmitoylation at Cys212 and Cys284.^[^ ^23^ ^]^ There is competition between these two modifications.^[^ ^31^ ^]^ The nNOS inhibitor increases protein palmitoylation, while treatment with palmitoylation inhibitors increases protein nitrosylation.^[^ ^31^, ^58^ ^]^ In our experiments, we used nNOS inhibitors or genetic silence of nNOS expression to reduce SNO‐gephyrin and improve anxiety‐like behaviors by increasing the membrane expression of GABA_A_R γ2. The reduction of gephyrin nitrosylation may also promote the aggregation of GABA_A_R at synaptic sites by increasing the palmitoylation of gephyrin, thereby improving anxiety‐like behaviors. Our previous study could not assess the relative contributions of gephyrin nitrosylation and palmitoylation to GABA_A_R aggregation at synaptic sites. However, the nitrosylation of gephyrin is considered to have a stronger impact on GABA_A_R cluster under current experimental conditions, according to the results of co‐immunoprecipitation, which reveal enhanced binding of gephyrin with GABA_A_R γ2. Further experiments are still required in the future, such as evaluating the relative magnitude of changes in gephyrin nitrosylation and palmitoylation in high anxiety rats.

As an intercellular messenger, NO participates in many processes in the central and peripheral nervous system, including learning, memory, anxiety, sleep, energy homeostasis, motor control, reproductive activity and cerebral blood flow regulation.^[^ ^51^, ^59^ ^]^ The L‐arginine‐dependent NOS pathway is generally regarded as the main source of endogenous NO formation.^[^ ^60^ ^]^ There are three isozymes of nitric oxide synthase, including nNOS that is mainly present in the nervous system, including cerebellum, striatum, cerebral cortex, and hippocampus,^[^ ^59^ ^]^ iNOS that is present in macrophages, liver cells, and glial cells, and eNOS that is mainly present in vascular endothelial cells. In this study, we presented evidence that nNOS was highly expressed in the BLA, and the level of nNOS was elevated in HA rats, while the levels of eNOS and iNOS were not changed. Knockdown of nNOS attenuated S‐nitrosylation of gephyrin and produced anxiolytic effects by increasing GABA_A_R trafficking. In addition, this effect can be mimicked by specific inhibitors of nNOS. In contrast, overexpression of nNOS in the BLA exacerbated anxiety‐like behaviors by reducing the surface expression of GABA_A_R and abolished the anxiolytic effect of physical exercise, indicating that physical exercise exerts anxiolytic effects through nNOS‐mediated gephyrin S‐nitrosylation. Since the increase in BDNF also participate in nNOS‐related emotional regulation,^[^ ^61^ ^]^ we could not exclude the potential role of BDNF in this process.

In summary, we identified an essential role of SNO‐gephyrin‐mediated inhibitory neurotransmission in anxiety‐like behaviors, which could be alleviated by wheel‐running exercise. In addition, selective inhibition of nNOS activity or site‐directed mutagenesis of S‐nitrosylated cysteine residues in gephyrin reduced anxiety‐like behaviors. We also demonstrated that physical exercise rescued the impairment in GABAergic transmission at least in part by reducing the expression of SNO‐gephyrin, ultimately improving anxiety‐like behaviors, revealing a previously unknown pathway by which exercise relieved anxiety‐like behaviors. Our study mainly fills the gap of post‐translational changes in proteins in the brain after exercise and expands the neurobiological mechanisms underlying the exercise‐dependent relief of anxiety. The finding that SNO‐gephyrin is downregulated by exercise to alleviate anxiety not only provides a potential target for the prevention and intervention of anxiety, but also provides clues for the development of more effective exercise prescriptions for the treatment of anxiety and mood disorders. Running exercise therapy has the potential to be further developed as a supplement for mental health.

## 4 Experimental Section

### Animals

Male Sprague Dawley (SD) rats (weight 200–250 g) used in this study were purchased from Hunan Silaike Jingda Laboratory Animal Corporation Ltd. (Changsha, China). The animals were raised at a constant temperature (22 ± 2 °C), a humidity of 50% ± 10%, and a light‐dark cycle of 12 h to obtain water and food freely. All experimental programs were conducted in accordance with the guidelines for the care and use of experimental animals of the National Institutes of Health, and were approved by the Animal Welfare Committee of Huazhong University of Science and Technology.

### Elevated Plus Maze Test

All behavioral tests were performed in adult rats according to this previous work.^[^ ^8^ ^]^ The EPM consists of two open arms and two closed arms (50 cm × 10 cm each) and a central region (10 cm × 10 cm). The closed arms were surrounded by a 50 cm high black wall. For behavioral test, rats were placed in the central area with their heads facing to the closed arms and allowed to explore freely for 5 min. ANY‐Maze behavioral tracking system (Stoelting Co, USA) was used to track, and record the number of times each rat enters the open arm, the time spent and the movement distance. The rats were divided into LA, IA and HA group according to the total time they stayed in the open arm in the EPM test. The rats who spent more than 60% of the time in the open arm were in the LA group, those who spent between 10% and 60% of the time in the open arm were in the IA group, and those who spent less than 10% of the time in the open arm were in the HA group.

### Open Field Test

The OFT was monitored in a plastic arena (100 cm (w) × 100 cm (d) × 40 cm (h)). Rats were placed gently in the central area and explored freely for 5 min. Then the ANY‐Maze system was used to record the number, time and distance of each rat entering the central area.

### Chronic Corticosterone Exposure Model

SD rats were randomly divided into control group and corticosterone (CORT) exposure group, with 8 rats in each group. The control group drank water containing vehicle (0.45% hydroxypropyl‐β‐cyclodextrin, Aladdin Reagent, Shanghai, China), and the corticosterone‐exposed group drank corticosterone (35 mg ml^−1^, Sigma–Aldrich, St. Louis, USA) dissolved in vehicle for 30 days.

### Detection of Protein S‐Nitrosylation

Protein S‐nitrosylation (SNO) was tested with biotin‐switch method (Figure S11, Supporting Information). The detection method was the same as described previously with minor modifications.^[^ ^8^, ^62^, ^63^ ^]^ The BLA slices were collected and homogenized in RIPA lysates (50 mM Tris‐HCl, pH 7.4, 1% NP‐40, 0.5% Na‐deoxycholate, 0.1% sodium dodecyl sulfate (SDS), 150 mM NaCl, 2 mM EDTA and 50 mM NaF) containing protease inhibitors (Roche, Basel, Switzerland) and phosphatase inhibitors (Sigma–Aldrich, St. Louis, USA). The lysate was centrifuged at 12 000 g for 15 min at 4 °C. The protein supernatant was collected, and the concentration was determined for each sample. 500 µg supernatant was mixed with 4 times volume of blocking buffer (9 volume (vol.). HEN buffer [250 mM HEPES pH7.7, 1 mM EDTA, 0.1 mM neocuproine] + 1 vol. 2.5% SDS + 1/100 vol. 20 mM methyl methanethiosulfonate (MMTS)) and shake slowly at 50 °C to block free thiols. After the reaction, the samples were centrifuged at 12 000 g for 20 min at 4 °C. The precipitate was redissolved in the HENS buffer (HEN buffer + 2.5% SDS), and 4 times the volume of pre‐cooled acetone was added to precipitate it, and it was allowed to be put at −20 °C for 20 min, and then centrifuged at 12 000 g at 4 °C for 20 min to remove excess MMTS (64 306, Sigma–Aldrich, St. Louis, USA). To completely remove remaining MMTS, this step was repeated three times. After the last precipitation, the pellet was redissolved in HENS buffer and split equally in two, one of which was supplemented with 100 mM ascorbate (Sigma–Aldrich, St. Louis, USA)^[^ ^64^ ^]^ and *N*‐(6‐(biotinamido) hexyl)−3‐(2‐pyridyldithio)‐propionamide (HPDP)‐biotin (0.2 mM) (21 341, Thermo Fisher Scientific, Waltham, USA) to reduce SNO and label it with HPDP‐biotin. Another aliquot was made without ascorbate and only with HPDP‐biotin, samples not reduced by ascorbic acid were used as the negative control. All samples were incubated at 37 °C for 2 h in a dark environment. After incubation, pre‐cooled acetone precipitation was added to remove the remaining ascorbic acid and HPDP‐biotin, and this step was repeated twice. Finally, the precipitate was redissolved with HENS buffer.

To detect protein S‐nitrosylation, the precipitated solution was divided equally into two parts. A portion was directly added to the SDS‐PAGE loading buffer as “total”. The other part was purified with streptavidin‐agarose (S1638, Sigma–Aldrich, St. Louis, USA). After repeated purification 10 times, it was washed 3 times with HENS buffer. The biotinylated protein was denatured by adding SDS‐PAGE loading buffer and eluted at 95 °C for 30 min, as “SNO”, and the level of S‐nitrosylation was analyzed by western blotting with a specific antibody.

### Detection of Protein S‐Palmitoylation

Protein S‐Palmitoylation (Pal) was tested with acyl‐biotin exchange assay. The detection method was the same as described previously with minor modifications.^[^ ^8^, ^31^ ^]^ The BLA slices were collected and homogenized in RIPA lysates containing protease inhibitors and phosphatase inhibitors. Extracts were sonicated briefly and treated with 20 mM MMTS for 50 min at 50 °C. Proteins were precipitated with acetone and labeled with biotin‐HPDP in buffer containing 0.70 M hydroxylamine (159 417, Sigma, Sigma–Aldrich, St. Louis, USA), pH 7.4 for 2 hours at room temperature. Add pre‐cooled acetone precipitate to remove remaining hydroxylamine and HPDP biotin. Finally, the precipitate was redissolved in HENS buffer, and the protein solution with hydroxylamine was divided into two parts. A portion solution was directly added to the SDS‐loading buffer as “total”. The other part was purified with streptavidin‐agarose. After repeated purification 10 times, it was washed 3 times with HENS buffer. The biotinylated protein was denatured by adding SDS‐loading buffer and eluted at 95 °C for 30 min, as “Pal”. Biotinylated proteins were separated by SDS PAGE, and analyzed by western blot.

### Co‐Immunoprecipitation

The BLA slices were lysed with NP‐40 buffer (50 mM Tris (pH 7.4), 150 mM NaCl, 1% NP‐40) containing protease inhibitors. 500 µg of total protein was incubated with mouse anti‐gephyrin antibody or mouse serum control IgG overnight at 4 °C. The complexes containing the target protein and interacting proteins are then immunoprecipitated by centrifugation using protein A/G agarose beads (sc‐2003, Santa Cruz Bio technology, Dallas, USA). The immune complexes were washed three times with lysis buffer, aspirated to dryness with a syringe, resuspended in 2× SDS sample buffer, boiled at 95 °C for 5 min, and analyzed by western blot.^[^ ^65^, ^66^ ^]^

### Western Blot

The BLA tissues were collected and homogenized in RIPA lysates containing protease inhibitors and phosphatase inhibitors. The lysate was centrifuged at 12 000 g for 15 min at 4 °C, and the protein supernatant was mixed with 4 × loading buffer and heated at 95 °C for 5 min. Lysates were separated by SDS‐polyacrylamide gel electrophoresis (PAGE) and transferred to nitrocellulose membranes. Transferred membranes were incubated overnight at 4 °C with primary antibodies: GABA_A_R γ2 (1:1000, 224 003, Synaptic Systems, Göttingen, Germany), GABA_A_R α1 (1:1000, ab33299, Abcam, Cambridge, USA), GABA_A_R α2 (1:1000, ab72445, Abcam, Cambridge, USA), GABA_A_R α3 (1:1000, 224 302, Synaptic Systems, Göttingen, Germany), GABA_A_R β2 (1:1000, ab186875, Abcam, Cambridge, USA), nNOS (1:1000, 61–7000, Thermo Fisher Scientific, Waltham, USA), iNOS (1:1000, 18985‐1‐AP, Proteintech, Chicago, USA), gephyrin (1:1000, 610 584, BD, USA), Caspase‐3 (1:1000, #9661, Cell Signaling Technology, Danvers, USA), RIP3 (1:500, SC‐374639, Santa Cruz Biotechnology, California, USA), Bcl‐2 (1:200, SC‐7382, Santa Cruz Biotechnology, California, USA), Caspase‐1 (1:1000, 06‐503‐I, Millipore, Bedford, USA), β‐actin (1:3000, sc‐47778, Santa Cruz Biotechnology, California, USA). After the membrane was washed 3 times in TBS‐Tween 20, it was incubated with horseradish peroxidase‐conjugated secondary antibody for 1 h at room temperature. After continuing to wash with TBS‐Tween 20, visualization was performed using MicroChemi (DNR Bio‐imaging systems, Jerusalem, Israel). The optical density of the immunoblots were measured with ImageJ software (NIH, MD, USA). All results were expressed as a normalized percentage of control.

For surface protein extraction, the BLA slices were cross‐linked with 1.0 mg ml^−1^ sulfo‐*N*‐hydroxysuccinimide (NHS)‐long‐chain‐biotin (21 335, Thermo Fisher Scientific, Rockford, USA) for 90 min at 4 °C, and the reaction was terminated with 100 mM glycine. After the reaction, the protein supernatant was prepared with RIPA lysate, and measured the concentration of protein supernatant of each sample. Protein supernatants were incubated with NeutrAvidin coupled‐agarose beads (29 201, Thermo Fisher Scientific, Rockford, USA) overnight at 4 °C, then mixed with 2 × loading buffer and heated at 95 °C for 5 min.

### Quantitative Real‐Time PCR

Total RNA was extracted from the BLA sections using TRIzol reagent (Life Technologies, NY, USA). 500 ng of total RNA was reverse transcribed into cDNA using the RevertAid First Strand cDNA synthesis kit (Fermentas, Thermo Fisher Scientific, Canada). Quantitative real‐time PCR was performed on the StepOnePlusTM Real‐Time PCR System (Applied Biosystems, Foster City, CA) using the SYBR Premix Ex Taq kit (Takara Bio Inc., Tokyo, Japan). The nNOS amplification conditions were 95 °C for 30 s, 95 °C for 5 s, 60 °C for 30 s, and 40 cycles. The eNOS amplification conditions were 95 °C for 30 s, 95 °C for 5 s, and 59 °C for 30 s, 40 cycles. The ΔΔCt value was used for gene expression analysis, and the target gene expression was normalized to the glyceraldehyde‐3‐phosphate dehydrogenase (GAPDH) mRNA level. Primer sequences were used as follows (5′ to 3′): nNOS, GGAACCCTTGCGTTTCTTTCC (forward), AGGAGACGCTGTTGAATCGG (reverse); eNOS, CTGCCAACGTGGAGATCACT (forward), GGGGGCAGAGTGAAGAGTTC (reverse); GAPDH, ATGGTGAAGGTCGGTGTG (forward), CATTCTCGGCCTTGACTG (reverse).

### Electrophysiological Recording

Male SD rats (body weight 200–250 g) were anesthetized with pentobarbital sodium (40 mg kg^−1^) by intraperitoneal injection (*i.p*.), then decapitated, and the brains were cut into the BLA containing coronal slices. mIPSCs were recorded in the BLA slices using whole‐cell patch‐clamp recording. Rats were perfused with ice‐cold oxygenated cutting solution that contained (mM): sucrose 209, sodium L‐ascorbate 11.6, sodium pyruvate 3.1, glucose 22, NaH_2_PO_4_ 1.25, MgSO_4_•7H_2_O 4.9 and NaHCO_3_ 26, pH 7.4, osmolarity 300 mOsm. The BLA slices (300 µm thick) were cut using a vibrating blade microtome (VT1000S, Leica, Wetzlar, Germany) in cutting solution. Incubate the brain slices in artificial cerebrospinal fluid (ACSF) and recover at 28 °C for at least 1 h. ACSF (mM): NaCl 119.0, MgSO_4_ 1.3, KCl 3.5, NaH_2_PO_4_ 1.0, NaHCO_3_ 26.2, CaCl_2_ 2.5 and glucose 11.0, pH 7.4, osmolarity 300 mOsm. After recovering for 1 h, the BLA slices were transferred to the recording chamber and continuously perfused with ACSF at a rate of 2 ml min^−1^. The patch pipette (4–6 MΩ) was filled with an internal solution containing (mM): K‐gluconate 140, NaCl 8, MgCl_2_ 2, EGTA 1, HEPES 10, Mg‐ATP 2 and Na‐GTP 0.3, pH 7.2, osmolarity 300 mOsm. The mIPSCs were recorded in the BLA in voltage‐clamp mode at ‐70 mV. Glutamate receptor blockers 6, 7‐dinitroquinoline, 2, 3‐dione (CNQX, 20 µM), tetrodotoxin (TTX, 1 µM) and DL‐2‐amino‐5 phosphonovalerate (AP‐5, 30 µM) were added to extracellular solution during recording. All data was obtained by pClamp10 (Axon instruments, Molecular Devices, San Jose, CA) and Multiclamp 700B (Molecular Devices, Sunnyvale, CA) amplifier software, and the signal was filtered at 5 kHz and digitized at 10 kHz.

### Virus Injection

Rats were anesthetized with sodium pentobarbital (40 mg kg^−1^, intraperitoneally) and fixed on a stereotaxic apparatus. For genetic knockdown of nNOS, Lentivirus (LV)‐shRNA‐nNOS (target sequence: AACAGCCAAAGCAGAGATGAA) or shRNA‐eGFP were microinjected within the BLA (anterior: – 2.9 mm; medial‐lateral: ± 5.0 mm; dorsal‐ventral: – 8.5 mm; relative to bregma). The final titer of LV‐shRNA‐nNOS was 2.0 × 10^9^ GC mL^−1^. In order to overexpress nNOS, LV‐nNOS or LV‐eGFP were microinjected into the BLA. The final titer of LV‐nNOS was 2.0 × 10^9^ GC mL^−1^. For overexpression of gephyrin, LV‐Gphn^C212A^, LV‐Gphn^C284A^ and LV‐eGFP were transduced into the BLA by microinjection. The final titer of LV‐Gphn^C212A^ was 2.0 × 10^9^ GC mL^−1^. The final titer of LV‐Gphn^C284A^ was 3.0 × 10^9^ GC mL^−1^. All viruses were purchased from GeneChem Company (Shanghai, China). It was injected with 1 µL per side and the injection rate was 0.1 ml min^−1^.

### Drug Infusion

A 22‐gauge stainless steel guide cannulas (RWD life Science, Shenzhen, China) was implanted into the BLA using a stereotaxic apparatus. Rats were required to recover for 7 days after the implantation procedure was completed. DTT (43 815, Sigma–Aldrich, St. Louis, USA), DETA‐NONOate (ab144627, Abcam, Cambridge, UK), GABA_A_R α3 peptide and KT5823 (ab120423, Abcam, Cambridge, UK) were dissolved in artificial cerebrospinal fluid (ACSF) before use, and 7‐NI (N7778, Sigma–Aldrich, St. Louis, USA) was dissolved ACSF containing 0.1% DMSO. The sequence for TAT control peptide was YGRKKRRQRRR‐COOH for rat GABA_A_R ɑ3 and the sequence for TAT‐pep‐F368N peptide YGRKKRRQRRRFNIVGTTYPIN‐COOH for rat GABA_A_R ɑ3 (Jier, Shanghai, China).^[^ ^8^ ^]^ These drugs, including DTT (1.0 mM),^[^ ^67^ ^]^ GABA_A_R α3 peptide (100 µM),^[^ ^8^ ^]^ 7‐NI (100 µM),^[^ ^68^ ^]^ DETA‐NONOate (100 µM),^[^ ^26^ ^]^ KT5823 (1 µM) and vehicle,^[^ ^69^ ^]^ were injected into the BLA 30 min before the behavioral test with 1 µL per side of the cannulas through a 5 µL syringe. SNAP was purchased from Sigma–Aldrich (N3398, St. Louis, USA).

### Statistical Analysis

All data were presented as mean ± SEM and analyzed by GraphPad 8.0 software (GraphPad, San Diego, USA). Statistical significance was tested using the unpaired Student's t‐test for comparison between two groups. One‐way analysis of variance (ANOVA) followed by Bonferroni's post hoc test was used for multiple group comparisons. For data comparisons of more than two groups, two‐way ANOVA followed by Bonferroni's post hoc test was used. *P* < 0.05 was considered significant. The statistical analyses and sample size applied for each experiment were indicated in the figure legends. The details were provided in the Table S1 (Supporting Information).

## Conflict of Interest

The authors declare no conflict of interest.

## Author Contributions

P.‐F.Y. wrote the manuscript and performed most of the experiments. T.‐L.N. participated in some western blot detection. X.‐N.S. and L.‐X.X. performed the virus injection. M.C. participated in the supervision for the experiments. L.‐H.L. conceived the project and designed the experiments. J.‐G.C. and F.W. designed the project, revised the manuscript and supported funding acquisition.

## Supporting information

Supporting Information

## Data Availability

The data that support the findings of this study are available from the corresponding author upon reasonable request.
