# Correction to “Wheel‐Running Exercise Alleviates Anxiety‐Like Behavior via Down‐Regulating S‐Nitrosylation of Gephyrin in the Basolateral Amygdala of Male Rats”

**DOI:** 10.1002/advs.202415925

**Published:** 2025-01-14

Yang PF, Nie TL, Sun XN, Xu LX, Ma C, Wang F, Long LH, Chen JG. Wheel‐Running Exercise Alleviates Anxiety‐Like Behavior via Down‐Regulating S‐Nitrosylation of Gephyrin in the Basolateral Amygdala of Male Rats. *Adv Sci (Weinh)*. **2024**, *11*(34): e2400205.

In the original version of Figure 4, Figure 4K is incorrectly identical to that in Figure 2E. This is an error that occurred during sub‐figure assembly. The correct figure is presented below.

Original Figure 4K


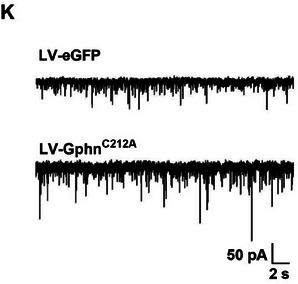


Corrected Figure 4K


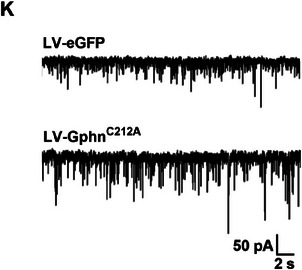


We have carefully reexamined all figures in the main document and Supporting Information, and we are confident that this correction does not impact the conclusions of our paper. We apologize for this error.

