# Supplementary material for: Wheel‐Running Exercise Alleviates Anxiety‐Like Behavior via Down‐Regulating S‐Nitrosylation of Gephyrin in the Basolateral Amygdala of Male Rats
Source: Adv Sci (Weinh). 2024 Jul 4;11(34):2400205. doi: 10.1002/advs.202400205 (PMC11425869; doi:10.1002/advs.202400205)
Supplement: Supplementary file 1 — Supporting Information [file ADVS-11-2400205-s001.docx]

Supporting Information
 **Wheel-Running** [**Exercise**](javascript:;) **Alleviates Anxiety-Like Behavior via Down-Regulating** **S-Nitrosylation of Gephyrin in the Basolateral Amygdala of Male Rats**

Ping-Fen Yang^1,2^, Tai-Lei Nie^1,2^, Xia-Nan Sun^1,2^, Lan-Xin Xu^1,2^, Cong Ma^3^, Fang Wang^1,2,4^, Li-Hong Long^1,2,4,*^, and Jian-Guo Chen^1,2,4,*^

^1^State Key Laboratory for Diagnosis and Treatment of Severe Zoonotic Infectious Diseases, Department of Pharmacology, School of Basic Medicine, Tongji Medical College, Huazhong University of Science and Technology, 430030, Wuhan, China

^2^The Key Laboratory for Drug Target Researches and Pharmacodynamic Evaluation of Hubei Province, 430030, Wuhan, China

^3^Key Laboratory of Molecular Biophysics of the Ministry of Education, College of Life Science and Technology, Huazhong University of Science and Technology, Wuhan, China.

^4^Hubei Shizhen Laboratory, 430030, Wuhan, China

^*^Corresponding authors: [chenj@mails.edu.cn](mailto:chenj@mails.edu.cn); longlihong@hust.edu.cn

**Supplementary figures**

**
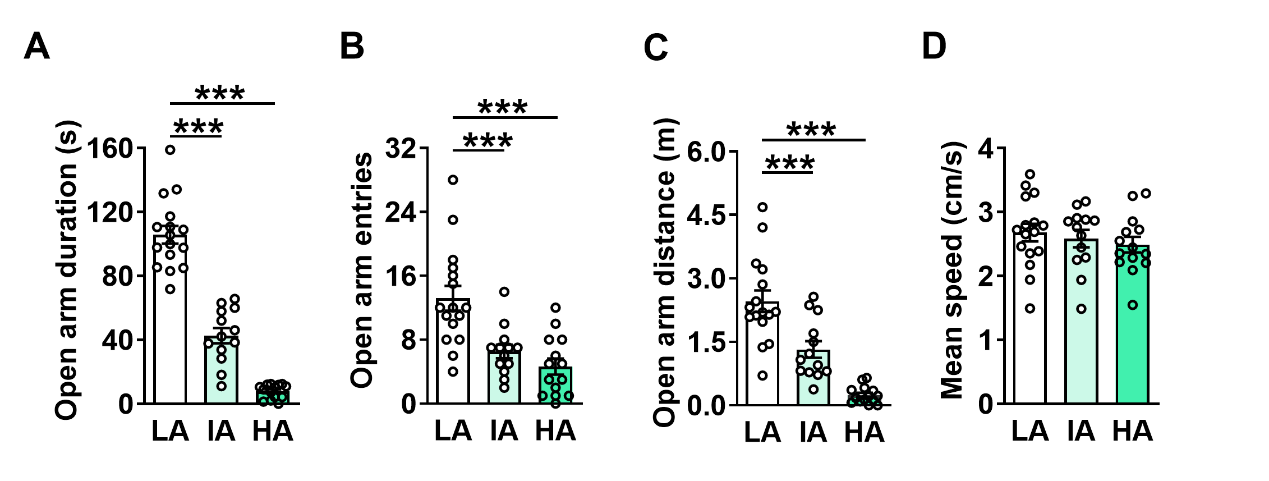
**

**Figure S1.** Rats are divided into high-anxiety group and low-anxiety group, according to the time spent in the open arm. A-C) The state of low, intermediate, and high-anxiety were observed according to the spent time A), entries B) and distance C) in open arm (*n* = 13-16 per group, one-way ANOVA followed by Bonferroni’s post hoc test). D) No significant difference in overall locomotor ability was observed (*n* = 13-16 per group, one-way ANOVA followed by Bonferroni’s post hoc test). All data are expressed as mean ± SEM, ****P* < 0.001. The statistical details can be found in Supplementary Table S1.


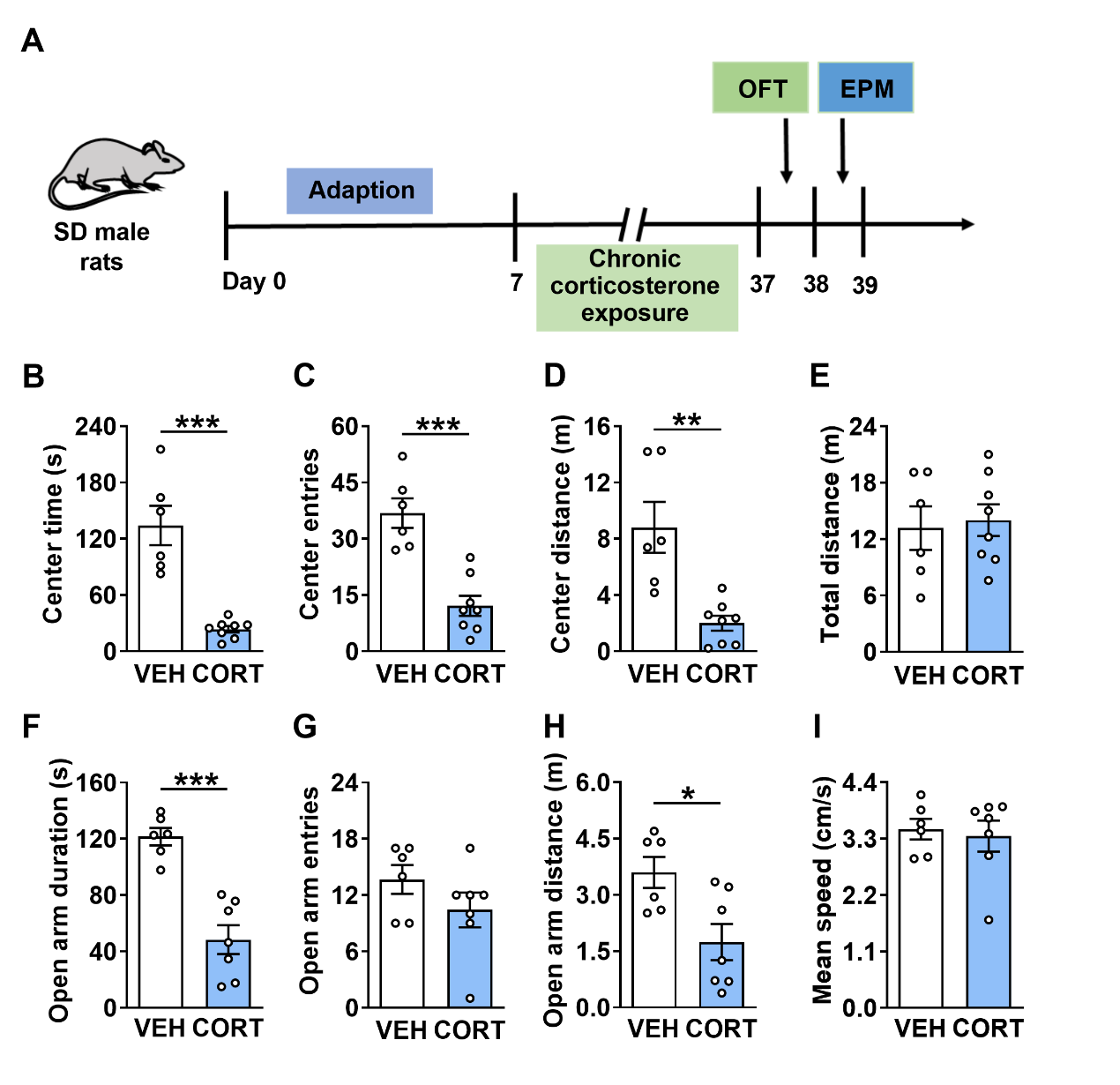


**Figure S2.** Chronic corticosterone exposure induces high-anxiety in rats. A) Schedule of chronic corticosterone exposure procedure. B-E) Chronic corticosterone exposure significantly reduced center time B), entries C), distance D) in OFT, but had no effect on the total distance traveled E) (*n* = 6-8 per group, Student’s *t-*test). F-I) Similar results have been observed in the EPM test. Chronic corticosterone exposure decreased the time and distance in open arm, no significant difference in the total distance traveled (*n* = 6-7 per group, Student’s *t-*test). All data are expressed as mean ± SEM, **P* < 0.05, ***P* < 0.01, ****P* < 0.001. The statistical details can be found in Supplementary Table S1.


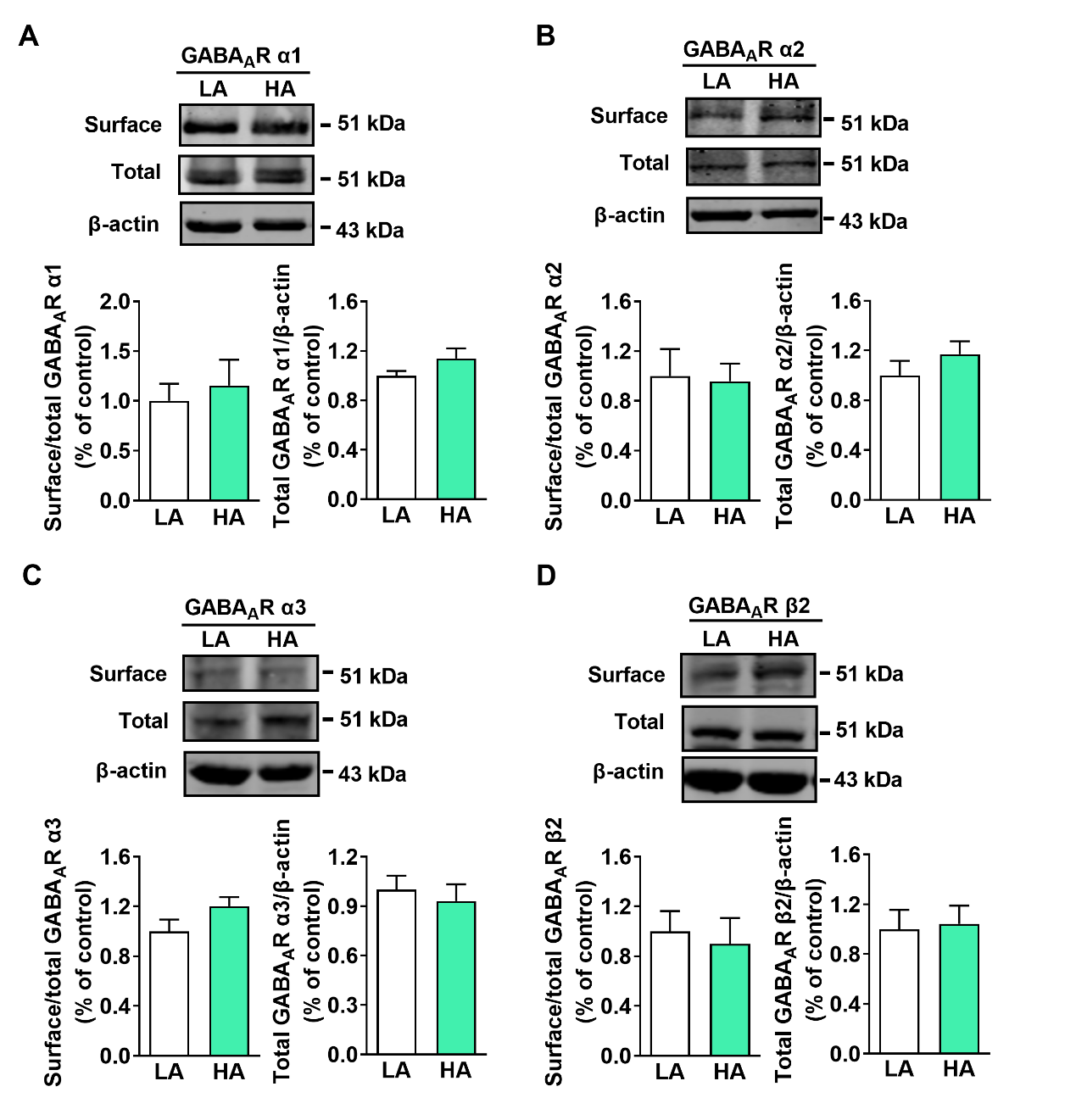


**Figure S3.** The unaltered surface expression of GABA_A_Rs in the BLA of HA rats. A) Quantification of sGABA_A_R α1 and tGABA_A_R α1 showing that there was no difference in LA and HA rats (*n* = 4 per group, Student’s *t-*test). B) Quantification of sGABA_A_R α2 and tGABA_A_R α2 showing that there was no difference in LA and HA rats (*n* = 4 per group, Student’s *t-*test). C) Quantification of sGABA_A_R α3 and tGABA_A_R α3 showing that there was no difference in LA and HA rats (*n* = 6 per group, Student’s *t-*test). D) Quantification of sGABA_A_R β2 and tGABA_A_R β2 showing that there was no difference in LA and HA rats (*n* = 6 per group, Student’s *t-*test). All data are expressed as mean ± SEM. The statistical details can be found in Supplementary Table S1.


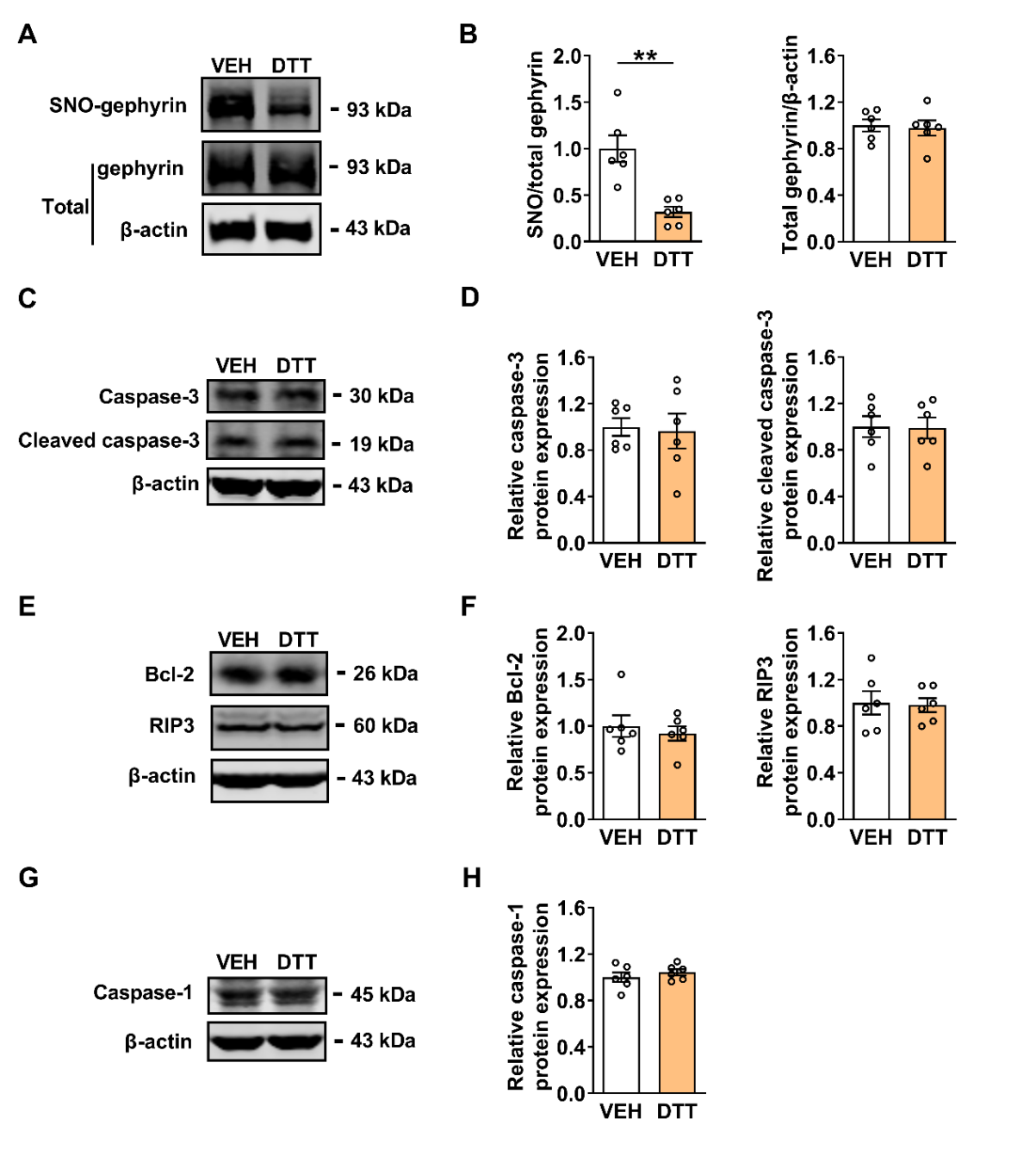


**Figure S4.** DTT significantly inhibits the expression of SNO-gephyrin. A) Representative western blot of SNO-gephyrin in the BLA of rat treated with DTT. B) DTT decreased the level of SNO-gephyrin, but not total gephyrin protein in BLA (*n* = 6 per group, Student’s *t-*test). C) Representative western blot of caspase-3 and cleaved caspase-3 in the BLA of rat treated with DTT. D) Quantitative analysis showed that DTT treatment had no effect on the protein level of caspase-3 and cleaved caspase-3 in the BLA of rat (*n* = 6 per group, Student’s t-test). E) Representative western blot of Bcl-2 and RIP3 in the BLA of rat treated with DTT. F) Quantitative analysis showed that DTT treatment had no effect on the protein level of Bcl-2 and RIP3 in the BLA of rat (*n* = 6 per group, Student’s t-test). G) Representative western blot of caspase-1 in the BLA of rat treated with DTT. H) Quantitative analysis showed that DTT treatment had no effect on the protein levels of caspase-1 in the BLA of rats (*n* = 6 per group, Student’s t-test). All data are expressed as mean ± SEM, ***P* < 0.01. The statistical details can be found in Supplementary Table S1.


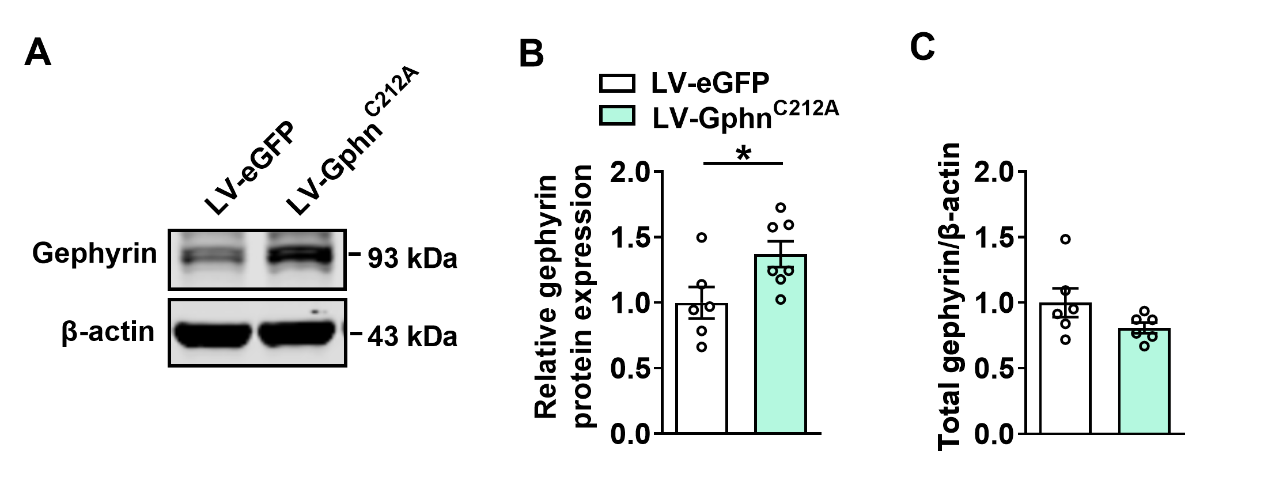


**Figure S5.** LV-Gphn^C212A^ has been successfully overexpressed in the BLA. A) Representative western blot of gephyrin in the BLA with LV-Gphn^C212A^ injection. B) The quantification showing that LV-Gphn^C212A^ was overexpressed in the BLA of rats (*n* = 6-7 per group, Student’s *t-*test). C) Western blot results of total gephyrin protein with LV-eGFP and LV-Gphn^C212^ groups (*n* = 6 per group, Student’s *t-*test). All data are expressed as mean ± SEM, **P* < 0.05. The statistical details can be found in Supplementary Table S1.


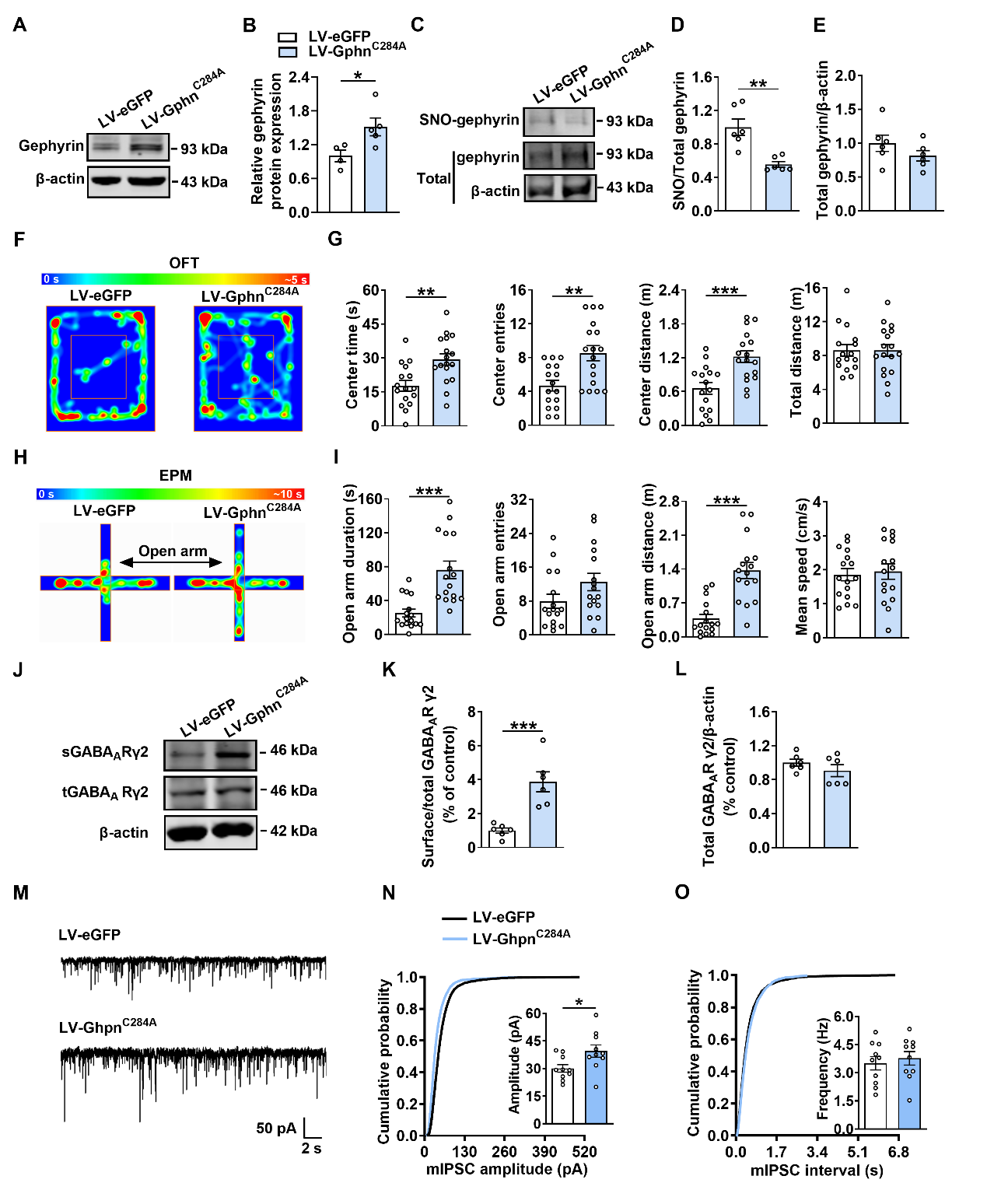


**Figure S6.** Inhibition of gephyrin S-nitrosylation by Cys284 mutation alleviates anxiety-like behaviors. A) Representative western blot of gephyrin in the BLA injected with LV-Gphn^C284A^. B) The quantification showing that the expression of gephyrin was increased with LV-Gphn^C284A^ in BLA (*n* = 4-5 per group, Student’s *t-*test). C) Representative western blot of SNO-gephyrin and total gephyrin protein from LV-eGFP and LV-Gphn^C284A^ groups. D-E) Western blot results showing that Cys284 mutation decreased the level of SNO-gephyrin, but not total gephyrin in the BLA (*n* = 6 per group, Student’s *t-*test). F, H) Representative heatmap from LV-eGFP and LV-Gphn^C284A^ rats in the OFT F) and EPM test H). G,I) Mutation of the SNO-gephyrin locus, Cys284, alleviated anxiety-like behaviors of rats in the OFT G) and EPM test I) (*n* = 16-17 per group, Student’s *t-*test). J) Representative western blot of sGABA_A_R γ2, tGABA_A_R γ2 in the BLA of rats injected with LV-Gphn^C284A^. K,L) Western blot results showing that mutation at Cys284 in SNO-gephyrin increased the ratio of sGABA_A_R γ2/tGABA_A_R γ2, but not tGABA_A_R γ2 expression in the BLA (*n* = 6 per group, Student’s *t-*test). M) Representative traces of mIPSCs in the BLA from LV-eGFP and LV-Gphn^C284A^ groups. Scale bar, 2 s, 50 pA. N,O) The amplitude of mIPSCs in LV-Gphn^C284A^ group increased N), but without change in the frequency O) (*n* = 10 cells from 4 rats per group, Student’s *t-*test). All data are expressed as mean ± SEM, **P* < 0.05, ***P* < 0.01, ****P* < 0.001. The statistical details can be found in Supplementary Table S1.


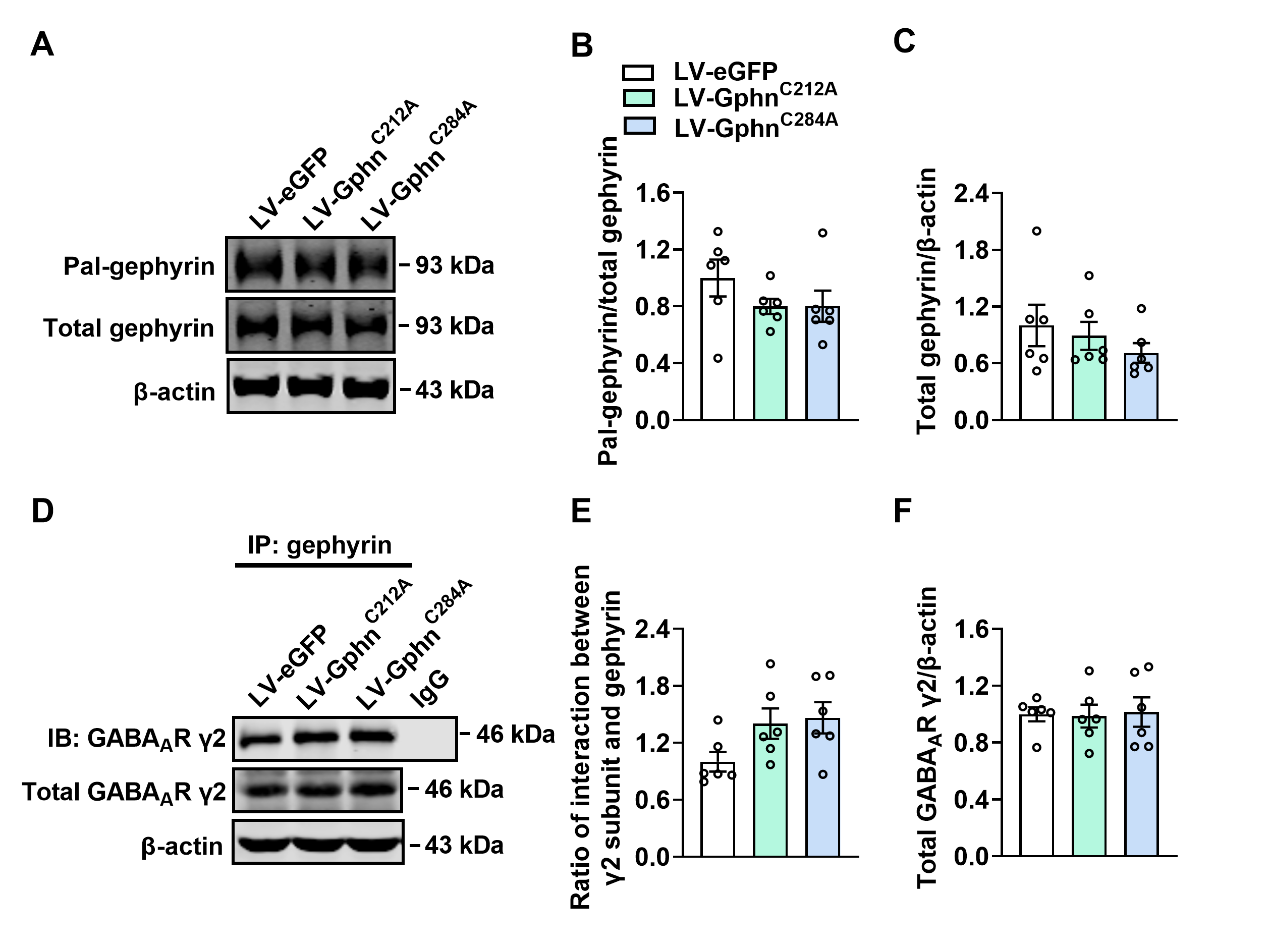


**Figure S7.** Mutating gephyrin nitrosylation sites at Cys212 or Cys284 has no obvious effect on gephyrin palmitoylation. A) Representative western blot of Pal-gephyrin in BLA after injection of LV-Gphn^C212A^ or LV-Gphn^C284A^. B) Pal-gephyrin in BLA showed a downward trend after injection of LV-Gphn^C212A^ or LVGphn^C284A^, but there was no statistical difference (*n* = 6 per group, one-way ANOVA followed by Bonferroni’s post hoc test). C) Treatment with LV-Gphn^C284A^ or LV-Gphn^C212A^ did not change the protein expression of total gephyrin in the BLA (*n* = 6 per group, one-way ANOVA followed by Bonferroni’s post hoc test). D) Representative western blot of GABA_A_R γ2 in BLA after injection of LV-Gphn^C212A^ or LV-Gphn^C284A^. E) Mutating the gephyrin nitrosylation at sites Cys212 or Cys284 has a tendency to increase the co-immunoprecipitation of GABA_A_R γ2 protein and gephyrin antibodies, but there is no significant statistical difference (*n* = 6 per group, one-way ANOVA followed by Bonferroni’s post hoc test). F) Mutating the gephyrin nitrosylation sites at Cys212 or Cys284 has no effect on the protein expression level of total GABA_A_R γ2 (*n* = 6 per group, one-way ANOVA followed by Bonferroni’s post hoc test). All data are expressed as mean ± SEM. The statistical details can be found in Supplementary Table S1.


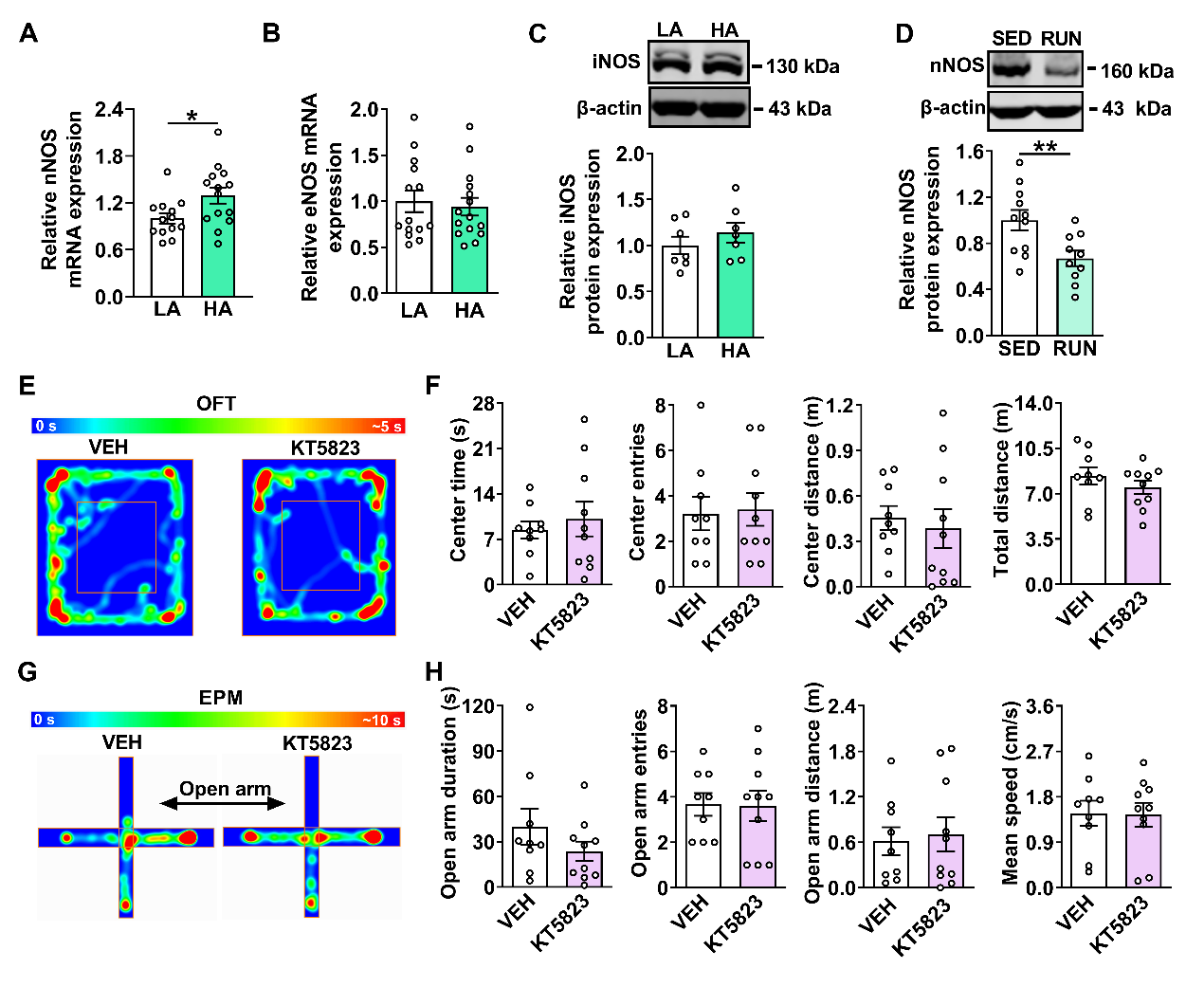


**Figure S8.** PKG inhibitor KT5823 has no effect on anxiety-like behaviors in male rats. A) *nNOS* mRNA expression in the BLA of LA and HA rats (*n* = 13-14 per group, Student’s *t-*test). B) *eNOS* mRNA expression in the BLA of LA and HA rats (*n* = 14-15 per group, Student’s *t-*test). C) Western blot results showing the protein expression of iNOS were unchanged in HA rats (*n* = 7 per group, Student’s *t-*test). D) Western blot results showing the protein expression of nNOS were decreased in RUN rats (*n* = 10-11 per group, Student’s *t-*test). E, G) Representative heatmap from vehicle and KT5823 (1 μM, 1μL per side) rats in the OFT E) and EPM test G). F, H) KT5823 treatment had no effect on anxiety-like behaviors in the OFT F) and EPM test H) (*n* = 9-10 per group, Student’s *t-*test). All data are expressed as mean ± SEM, **P* < 0.05, ***P* < 0.01. The statistical details can be found in Supplementary Table S1.

**
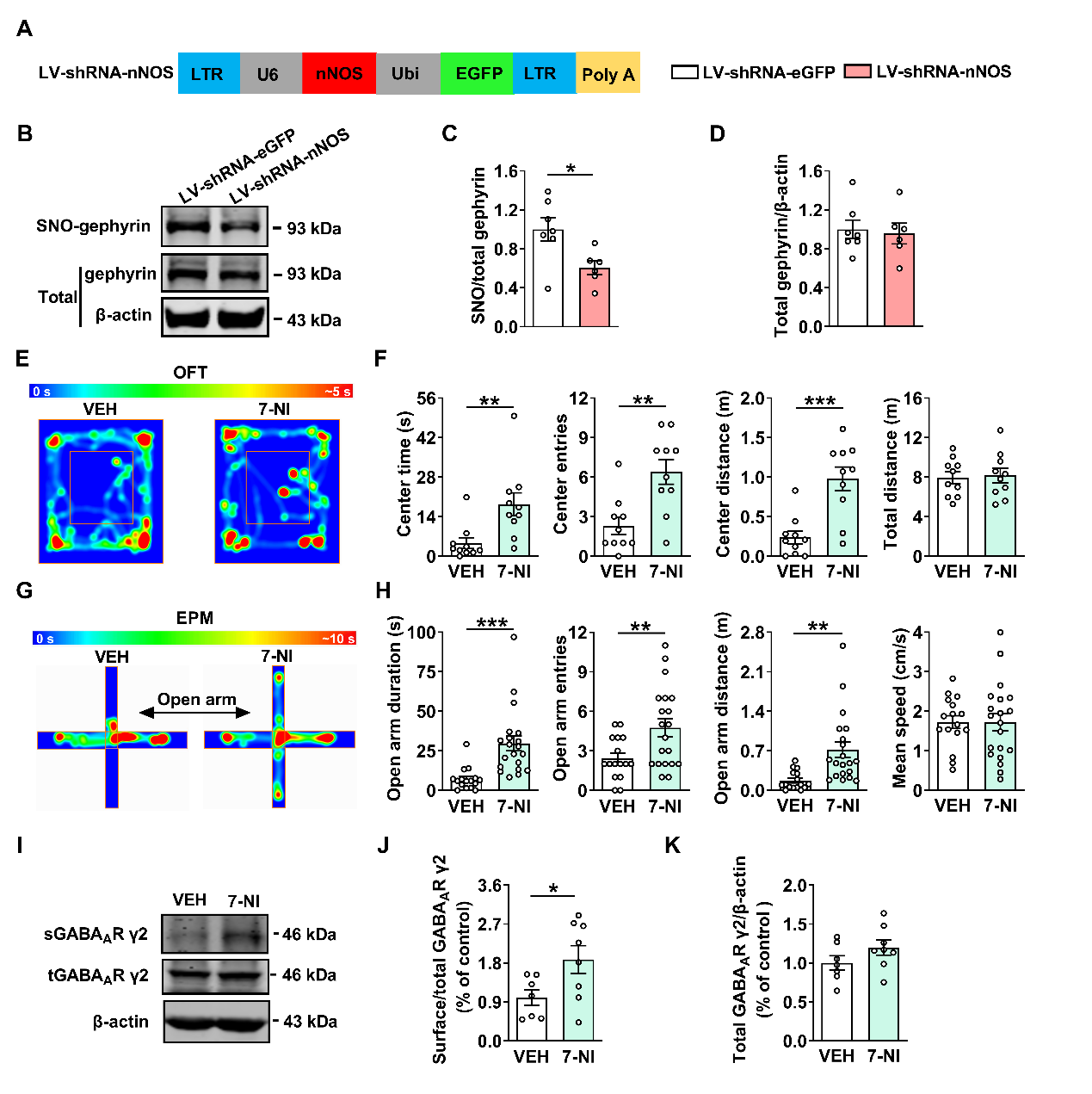
**

**Figure S9.** Specific inhibition on the activity of nNOS alleviates anxiety-like behaviors in male rats. A) Lentivirus (LV) vectors encoding enhanced green fluorescent protein (EGFP) and nNOS. B) Representative western blot of SNO-gephyrin and total gephyrin in the BLA of rats injected with LV-shRNA-nNOS. C,D) The quantification showing that LV-shRNA-nNOS decreased the expression of SNO-gephyrin, but not total gephyrin expression in the BLA (*n* = 6-7 per group, Student’s *t-*test). E,G) Representative heatmap from vehicle and 7-NI rats in the OFT E) and EPM test G). F,H) Selective inhibitor of nNOS significantly reduced anxiety-like behaviors of rats in OFT F) and EPM test H) (OFT: *n* = 10 per group, EPM: *n* = 16-20 per group, Student’s *t-*test). I) Representative western blot of sGABAAR γ2 and tGABA_A_R γ2 in the BLA with 7-NI treatment. J,K) Western blot results showing that treatment of 7-NI improved the expression sGABA_A_R γ2 protein, but not tGABA_A_R γ2 expression in the BLA (*n* = 7-8 per group, Student’s *t-*test). All data are expressed as mean ± SEM, **P* < 0.05, ***P* < 0.01, ****P* < 0.001. The statistical details can be found in Supplementary Table S1.


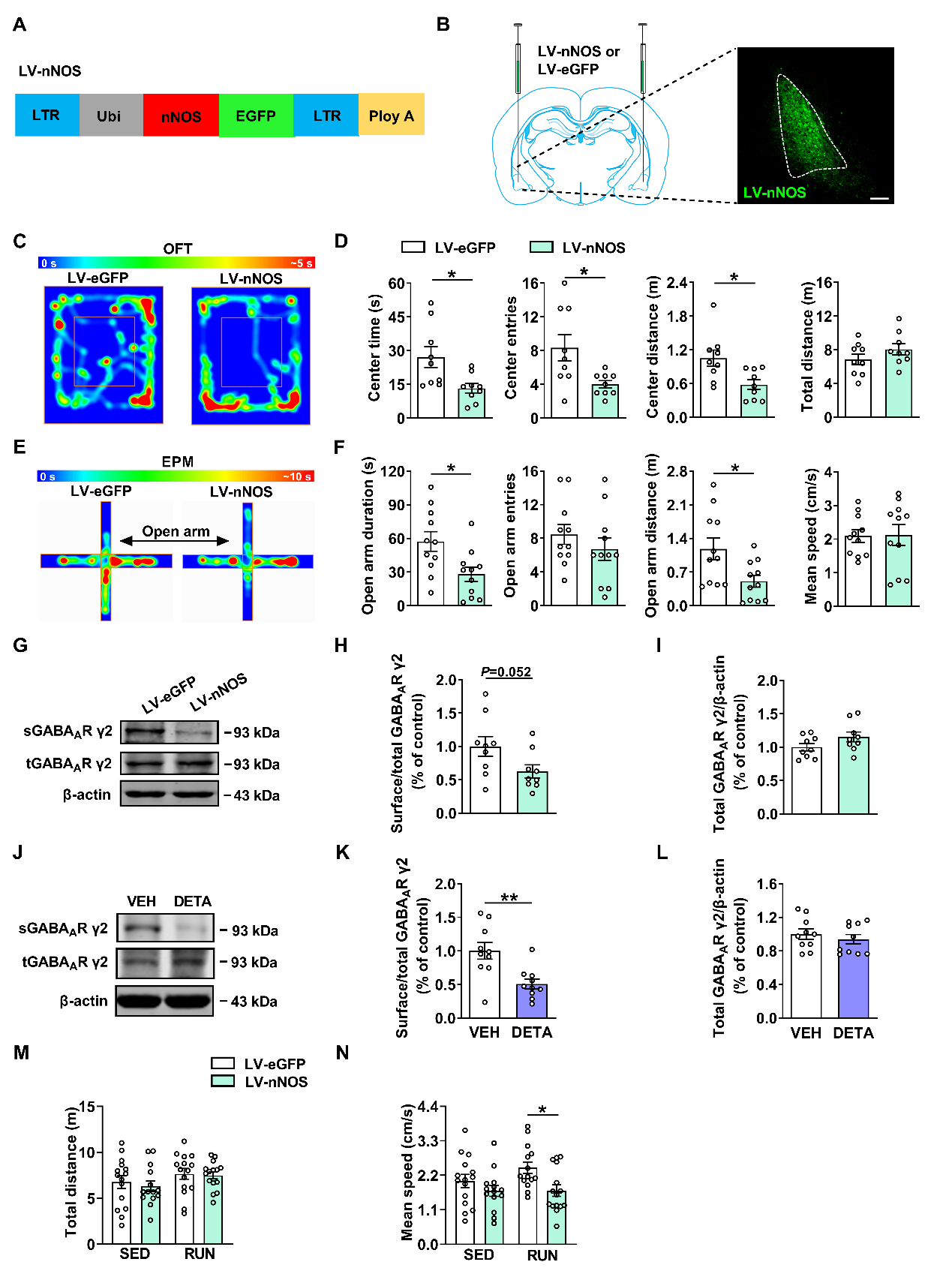


**Figure S10.** Overexpression of nNOS in BLA results in anxiety-like behaviors in rats. A) Lentivirus (LV) vectors encoding enhanced green fluorescent protein (EGFP) and nNOS. B) Representative image of LV-nNOS expression in one side of the BLA section two weeks after virus vector microinjection. green, LV-nNOS. Scale bar: 100 μm. C,E) Representative heatmap from LV-eGFP and LV- nNOS rats in OFT C) and EPM test E). D,F) nNOS overexpression increased anxiety-like behaviors compared with LV-eGFP in the OFT D) and EPM test F) (*n* = 9-11 per group, Student’s *t-*test). G) Representative western blot of sGABA_A_R γ2 and tGABA_A_R γ2 protein in the BLA treated with LV-nNOS. H,I) Western blot results showing that nNOS overexpression decreased the ratio of sGABA_A_R γ2/tGABA_A_R γ2 H), but not tGABA_A_R γ2 expression in the BLA I) (*n* = 9 per group, Student’s *t-*test). J) Representative western blot of sGABA_A_R γ2 and tGABA_A_R γ2 protein in the BLA treated with DETA-NONOate (DETA, 100 μM) treatment. K,L) Western blot results showing that DETA treatment decreased the ratio of sGABA_A_R γ2/tGABA_A_R γ2 K), but not tGABA_A_R γ2 expression in the BLA L) (*n* = 10 per group, Student’s *t-*test). M) LV-nNOS treatment did not affect the locomotor activity in OFT (*n* = 14-15 per group, two-way ANOVA followed by Bonferroni’s post hoc test). N) Treatment with LV-nNOS reduced the mean speed of movement in EPM of rats (*n* = 14-15 per group, two-way ANOVA followed by Bonferroni’s post hoc test). All data are expressed as mean ± SEM, **P* < 0.05, ***P* < 0.01. The statistical details can be found in Supplementary Table S1.

**
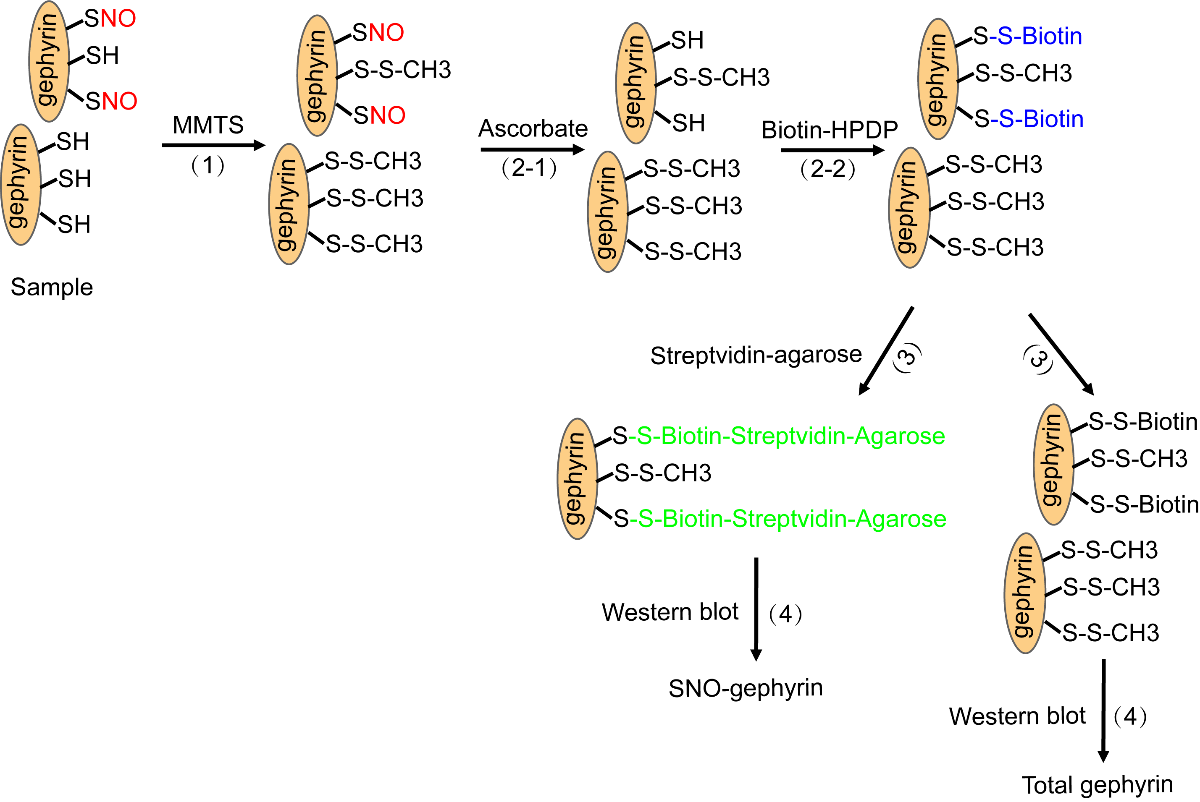
**

**Figure S11.** Detection of S-nitrosylation proteins by biotin-switch method. (1) After preparing tissue lysates, methyl methanethiosulfonate (MMTS) will be added and incubated to block free thiols. Then acetone precipitation will be used to remove unreacted MMTS. (2-1) Ascorbate selectively reduces S-nitrosothiols. (2-2) Label the reduced free thiols with N-(6-(biotinamido) hexyl)-3-(2-pyridyldithio)-propionamide (biotin-HPDP). (3) The biotinylated protein is divided into two parts. One part is directly added to the loading buffer and collected as total gephyrin. Another aliquot was purified with streptavidin-agarose as SNO-gephyrin. (4) Western blot analysis.


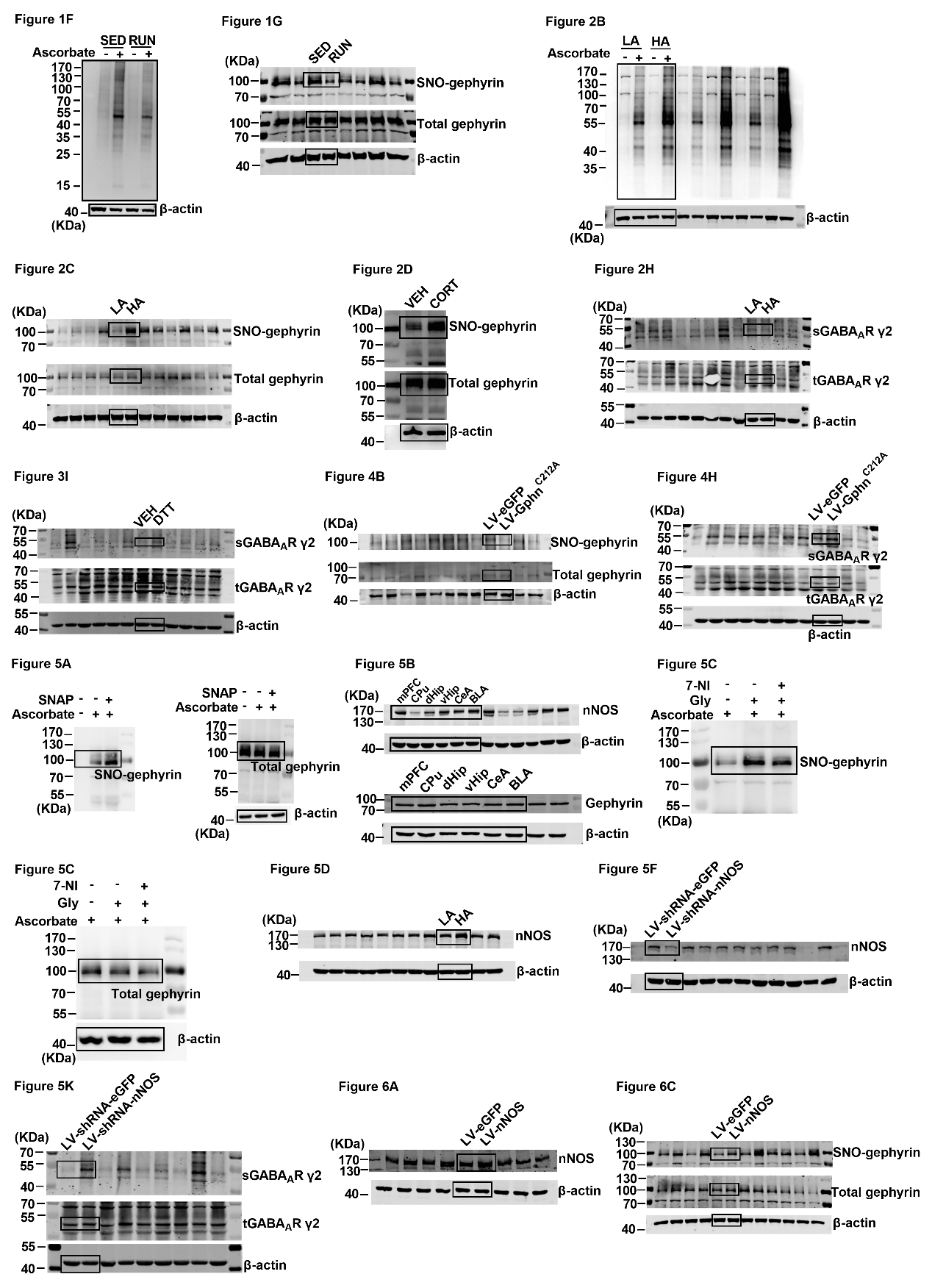


**Figure S12.** Original scans of key western blot in the figures

**
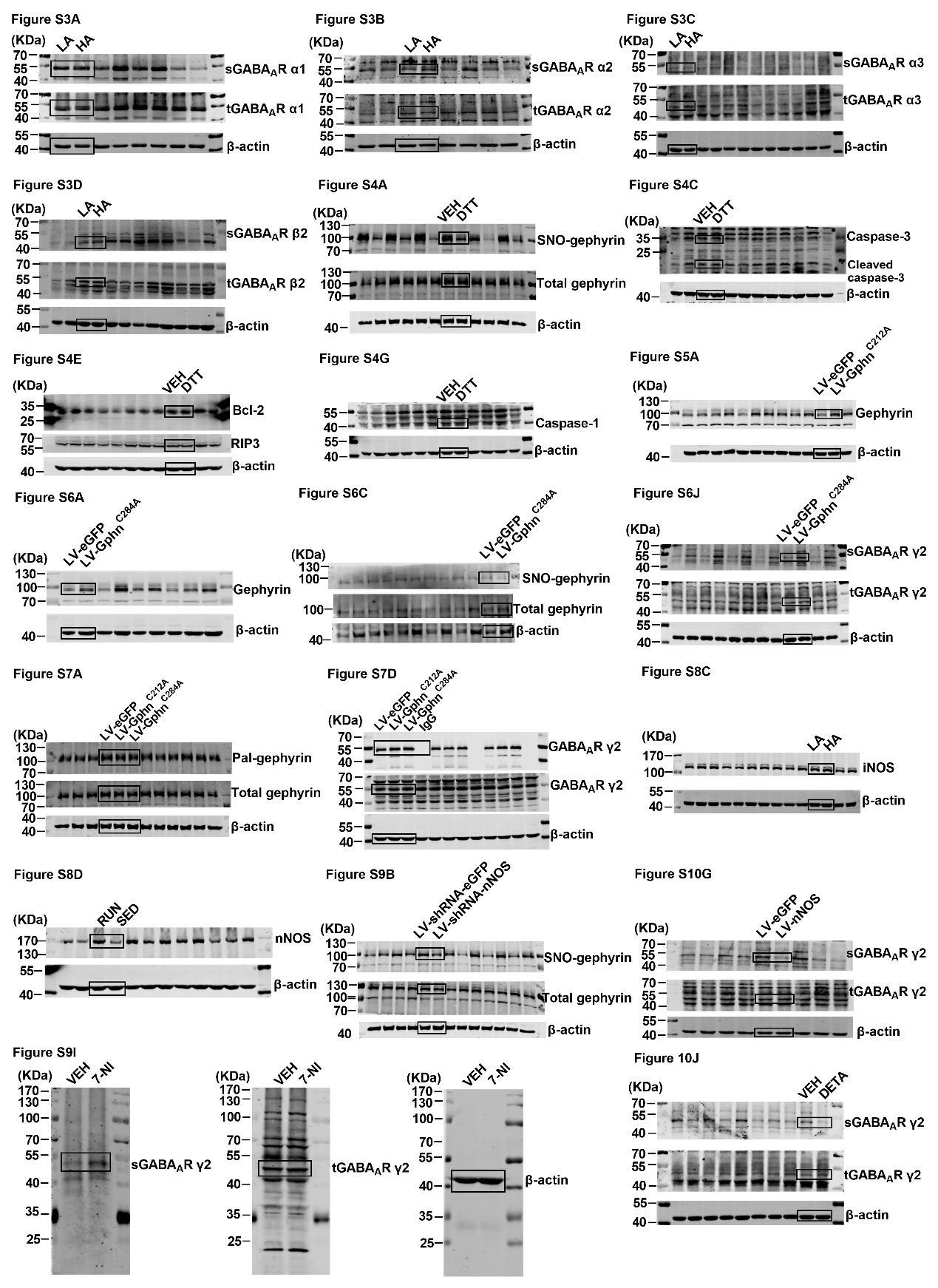
**

**Figure S13.** Original scans of key western blot in the supplementary figures

**Supplementary Table S1. Statistical analysis for Figures 1-6 and Figures S1-10.**

| **Figures and numbers of animals or cells** | **Statistical analysis** | ***Post hoc tests*** | **Mean ± SEM** |
| --- | --- | --- | --- |
| 1C: OFT  Center time:  SED (n = 10)  RUN (n = 10)  Center entries:  SED (n = 10)  RUN (n = 10)  Center distance:  SED (n = 10)  RUN (n = 10)  Total distance:  SED (n = 10)  RUN (n = 10) | Center time:  Unpaired t test  *t* = 4.083, *p* < 0.001  Center entries:  Unpaired t test  *t* = 3.934, *p* < 0.001  Center distance:  Unpaired t test  *t* = 4.359, *p* < 0.001  Total distance:  Unpaired t test  *t* = 1.694, *p* = 0.108 |  | Center time:  SED 13.930 ± 1.860  RUN 25.130 ± 2.016  Center entries:  SED 5.400 ± 0.957  RUN 11.000 ± 1.054  Center distance:  SED 0.789 ± 0.119  RUN 1.618 ± 0.149  Total distance:  SED 7.798 ± 1.130  RUN 10.506 ± 1.130 |
| 1E: EPM  Open arm duration:  SED (n = 10)  RUN (n = 9)  Open arm entries:  SED (n = 10)  RUN (n = 9)  Open arm distance:  SED (n = 10)  RUN (n = 9)  Mean speed:  SED (n = 10)  RUN (n = 9) | Open arm duration:  Unpaired t test  *t* = 3.165, *p* < 0.01  Open arm entries:  Unpaired t test  *t* = 1.530, *p* = 0.144  Open arm distance:  Unpaired t test  *t* = 3.414, *p* < 0.01  Mean speed:  Unpaired t test  *t* = 1.002, *p* = 0.331 |  | Open arm duration:  SED 43.740 ± 6.242  RUN 89.922 ± 13.762  Open arm entries:  SED 11.100 ± 2.030  RUN 16.222 ± 2.717  Open arm distance:  SED 1.082 ± 0.313  RUN 2.897 ± 0.440  Mean speed:  SED 2.535 ± 0.252  RUN 2.976 ± 0.371 |
| 1H: SNO-gephyrin protein expression in BLA  SED (n = 6)  RUN (n = 6)  Total gephyrin protein expression in BLA  SED (n = 6)  RUN (n = 6) | SNO-gephyrin protein expression in BLA  Unpaired *t* test  *t* = 8.473, *p* < 0.001  Total gephyrin protein expression in BLA  Unpaired *t* test  *t* = 0.878, *p* = 0.401 |  | SNO-gephyrin protein expression in BLA  SED 1.000 ± 0.058  RUN 0.430 ± 0.034  Total gephyrin protein expression in BLA  SED 1.000 ± 0.087  RUN 0.904 ± 0.067 |
| 1J: mIPSCs amplitude SED (n = 11)  RUN (n = 11) | Unpaired *t* test  *t* = 5.241, *p* < 0.001 |  | SED 26.770 ± 0.997  RUN 35.616 ± 1.362 |
| 1K: mIPSCs frequency  SED (n = 11)  RUN (n = 11) | Unpaired *t* test  *t* = 1.248, *p* = 0.227 |  | SED 4.611 ± 0.511  RUN 5.372 ± 0.332 |
| 2C: SNO-gephyrin protein expression in BLA  LA (n = 6)  HA (n = 6)  Total gephyrin protein expression in BLA  LA (n = 6)  HA (n = 6) | SNO-gephyrin protein expression in BLA  Unpaired *t* test  *t* = 2.264, *p* < 0.05  Total gephyrin protein expression in BLA  Unpaired *t* test  *t* = 0.068, *p* = 0.947 |  | SNO-gephyrin protein expression in BLA  LA 1.000 ± 0.246  HA 1.892 ± 0.308  Total gephyrin protein expression in BLA  LA 1.000 ± 0.122  HA 1.015 ± 0.176 |
| 2D: SNO-gephyrin protein expression in BLA  VEH (n = 6)  CORT (n = 6)  Total gephyrin protein expression in BLA  VEH (n = 6)  CORT (n = 6) | SNO-gephyrin protein expression in BLA  Unpaired *t* test  *t* = 2.269, *p* < 0.05  Total gephyrin protein expression in BLA  Unpaired *t* test  *t* = 0.047, *p* = 0.964 |  | SNO-gephyrin protein expression in BLA  VEH 1.000 ± 0.102  CORT 1.341 ± 0.110  Total gephyrin protein expression in BLA  VEH 1.000 ± 0.057  CORT 1.004 ± 0.070 |
| 2F: mIPSCs amplitude  LA (n = 11)  HA (n = 11) | Unpaired *t* test  *t* = 5.056, *p* < 0.001 |  | LA 32.512 ± 1.860  HA 22.452 ± 0.707 |
| 2G: mIPSCs frequency  LA (n = 11)  HA (n = 11) | Unpaired *t* test  *t* = 0.343, *p* = 0.735 |  | LA 3.710 ± 0.389  HA 3.883 ± 0.323 |
| 2H: sGABAAR γ2 protein expression in BLA  LA (n = 6)  HA (n = 6)  tGABAAR γ2 protein expression in BLA  LA (n = 6)  HA (n = 6) | sGABAAR γ2 protein expression in BLA  Unpaired *t* test  *t* = 2.344, *p* < 0.05  tGABAAR γ2 protein expression in BLA  Unpaired *t* test  *t* = 1.365, *p* = 0.202 |  | sGABAAR γ2 protein expression in BLA  LA 1.000 ± 0.134  HA 0.593 ± 0.110  tGABAAR γ2 protein expression in BLA  LA 1.000 ± 0.101  HA 1.422 ± 0.292 |
| 3B: OFT  Center time:  Control peptide (n = 10)  GABA_A_R α3 peptide (n = 9)  Center entries:  Control peptide (n = 10)  GABA_A_R α3 peptide (n = 9)  Center distance:  Control peptide (n = 10)  GABA_A_R α3 peptide (n = 9)  Total distance:  Control peptide (n = 10)  GABA_A_R α3 peptide (n = 9) | Center time:  Unpaired *t* test  *t* = 2.882, *p* < 0.05  Center entries:  Unpaired *t* test  *t* = 6.130, *p* < 0.001  Center distance:  Unpaired *t* test  *t* = 5.052, *p* < 0.001  Total distance:  Unpaired *t* test  *t* = 0.242, *p* = 0.812 |  | Center time:  Control peptide 23.520 ± 3.168  GABA_A_R α3 peptide 12.633 ± 1.849  Center entries:  Control peptide 8.400 ± 0.581  GABA_A_R α3 peptide 3.444 ± 0.556  Center distance:  Control peptide 1.361 ± 0.135  GABA_A_R α3 peptide 0.487 ± 0.103  Total distance:  Control peptide 7.736 ± 0.540  GABA_A_R α3 peptide 7.949 ± 0.705 |
| 3D: EPM  Open arm duration:  Control peptide (n = 10)  GABA_A_R α3 peptide (n = 9)  Open arm entries:  Control peptide (n = 10)  GABA_A_R α3 peptide (n = 9)  Open arm distance:  Control peptide (n = 10)  GABA_A_R α3 peptide (n = 9)  Mean speed:  Open arm distance:  Control peptide (n = 10)  GABA_A_R α3 peptide (n = 9) | Open arm duration:  Unpaired *t* test  *t* = 3.070, *p* < 0.01  Open arm entries:  Unpaired *t* test  *t* = 2.084, *p* = 0.053  Open arm distance:  Unpaired *t* test  *t* = 2.020, *p* = 0.060  Mean speed:  Unpaired *t* test  *t* = 0.526, *p* = 0.606 |  | Open arm duration:  Control peptide 61.980 ± 12.835  GABA_A_R α3 peptide 18.289 ± 4.545  Open arm entries:  Control peptide 8.200 ± 1.332  GABA_A_R α3 peptide 4.778 ± 0.894  Open arm distance:  Control peptide 1.472 ± 0.331  GABA_A_R α3 peptide 0.644 ± 0.226  Mean speed:  Control peptide 2.226 ± 0.157  GABA_A_R α3 peptide 2.407 ± 0.318 |
| 3F: OFT  Center time:  VEH (n = 11)  DTT (n = 11)  Center entries:  VEH (n = 11)  DTT (n = 11)  Center distance:  VEH (n = 11)  DTT (n = 11)  Total distance:  VEH (n = 11)  DTT (n = 11) | Center time:  Unpaired *t* test  *t* = 3.909, *p* < 0.001  Center entries:  Unpaired *t* test  *t* = 4.564, *p* < 0.001  Center distance:  Unpaired *t* test  *t* = 4.313, *p* < 0.001  Total distance:  Unpaired *t* test  *t* = 1.115, *p* = 0.278 |  | Center time:  VEH 10.836 ± 2.394  DTT 23.055 ± 2.009  Center entries:  VEH 4.455 ± 0.857  DTT 9.364 ± 0.651  Center distance:  VEH 0.586 ± 0.123  DTT 1.233 ± 0.086  Total distance:  VEH 6.943 ± 0.800  DTT 8.029 ± 0.557 |
| 3H: EPM  Open arm duration:  VEH (n = 12)  DTT (n = 12)  Open arm entries:  VEH (n = 12)  DTT (n = 12)  Open arm distance:  VEH (n = 12)  DTT (n = 12)  Mean speed:  VEH (n = 12)  DTT (n = 12) | Open arm duration:  Unpaired *t* test  *t* = 4.133, *p* < 0.001  Open arm entries:  Unpaired *t* test  *t* = 3.409, *p* < 0.01  Open arm distance:  Unpaired *t* test  *t* = 4.358, *p* < 0.001  Mean speed:  Unpaired *t* test  *t* = 0.731, *p* = 0.472 |  | Open arm duration:  VEH 23.933 ± 4.224  DTT 63.775 ± 8.667  Open arm entries:  VEH 3.917 ± 0.733  DTT 6.750 ± 0.392  Open arm distance:  VEH 0.453 ± 0.128  DTT 1.595 ± 0.229  Mean speed:  VEH 1.978 ± 0.184  DTT 2.165 ± 0.179 |
| 3J: sGABA_A_R γ2 protein expression in BLA after DTT treatment  VEH (n = 12)  DTT (n = 10) | Unpaired *t* test  *t* = 2.790, *p* < 0.05 |  | VEH 1.000 ± 0.104  DTT 1.424 ± 0.111 |
| 3K: tGABAAR γ2 protein expression in BLA after DTT treatment  VEH (n = 12)  DTT (n = 10) | Unpaired *t* test  *t* = 0.334, *p* = 0.742 |  | VEH 1.000 ± 0.064  DTT 0.970 ± 0.064 |
| 4C: SNO-gephyrin protein expression in BLA  LV-eGFP (n = 6)  LV-Gphn^C212A^ (n = 6) | Unpaired t test  *t* = 2.440, *p* < 0.05 |  | LV-eGFP 1.000 ± 0.182  LV-Gphn^C212A^ 0.507 ± 0.086 |
| 4E: OFT  Center time:  LV-eGFP (n = 17)  LV-Gphn^C212A^ (n = 17)  Center entries:  LV-eGFP (n = 17)  LV-Gphn^C212A^ (n = 17)  Center distance:  LV-eGFP (n = 17)  LV-Gphn^C212A^ (n = 17)  Total distance:  LV-eGFP (n = 17)  LV-Gphn^C212A^ (n = 17) | Center time:  Unpaired t test  *t* = 3.919, *p* < 0.001  Center entries:  Unpaired t test  *t* = 3.911, *p* < 0.001  Center distance:  Unpaired t test  *t* = 4.027, *p* < 0.001  Total distance:  Unpaired t test  *t* = 0.759, *p* = 0.453 |  | Center time:  LV-eGFP 11.765 ± 2.024  LV-Gphn^C212A^ 26.912 ± 3.292  Center entries:  LV-eGFP 4.529 ± 0.595  LV-Gphn^C212A^ 8.000 ± 0.659  Center distance:  LV-eGFP 0.548 ± 0.099  LV-Gphn^C212A^ 1.234 ± 0.139  Total distance:  LV-eGFP 7.810 ± 0.641  LV-Gphn^C212A^ 8.456 ± 0.559 |
| 4G: EPM  Open arm duration:  LV-eGFP (n = 17)  LV-Gphn^C212A^ (n = 17)  Open arm entries:  LV-eGFP (n = 17)  LV-Gphn^C212A^ (n = 17)  Open arm distance:  LV-eGFP (n = 17)  LV-Gphn^C212A^ (n = 17)  Mean speed:  LV-eGFP (n = 17)  LV-Gphn^C212A^ (n = 17) | Open arm duration:  Unpaired t test  *t* = 3.512, *p* < 0.01  Open arm entries:  Unpaired t test  *t* = 1.241, *p* = 0.224  Open arm distance:  Unpaired t test  *t* = 3.721, *p* < 0.001  Mean speed:  Unpaired t test  *t* = 0.339, *p* = 0.737 |  | Open arm duration:  LV-eGFP 19.865 ± 4.117  LV-Gphn^C212A^ 51.900 ± 8.139  Open arm entries:  LV-eGFP 4.765 ± 0.941  LV-Gphn^C212A^ 6.412 ± 0.936  Open arm distance:  LV-eGFP 0.510 ± 0.149  LV-Gphn^C212A^ 1.442 ± 0.201  Mean speed:  LV-eGFP 2.091 ± 0.162  LV-Gphn^C212A^ 2.184 ± 0.221 |
| 4I: sGABA_A_R γ2 protein expression in BLA  LV-eGFP (n = 6)  LV-Gphn^C212A^ (n = 6) | Unpaired *t* test  *t* = 2.250, *p* < 0.05 |  | LV-eGFP 1.000 ± 0.152  LV-Gphn^C212A^ 1.608 ± 0.224 |
| 4J: tGABA_A_R γ2 protein expression in BLA  LV-eGFP (n = 6)  LV-Gphn^C212A^ (n = 6) | Unpaired *t* test  *t* = 0.800, *p* = 0.442 |  | LV-eGFP 1.000 ± 0.074  LV-Gphn^C212A^ 0.912 ± 0.080 |
| 4L: mEPSCs amplitude  LV-eGFP (n = 9)  LV-Gphn^C212A^ (n = 9) | Unpaired *t* test  *t* = 4.400, *p* < 0.001 |  | LV-eGFP 29.704 ± 1.935  LV-Gphn^C212A^ 42.342 ± 2.123 |
| 4M: mEPSCs frequency  LV-eGFP (n = 9)  LV-Gphn^C212A^ (n = 9) | Unpaired *t* test  *t* = 1.318, *p* = 0.206 |  | LV-eGFP 5.280 ± 0.347  LV-Gphn^C212A^ 4.603 ± 0.379 |
| 5A: SNO-gephyrin protein expression in BLA after SNAP treatment  Control (n = 4)  SNAP (n = 4)  SNAP + Ascorbate (n = 4)  Total gephyrin protein expression in BLA after SNAP treatment  Control (n = 4)  SNAP (n = 4)  SNAP + Ascorbate (n = 4) | SNO-gephyrin protein expression in BLA after SNAP treatment  One-way ANOVA SNAP: F (2, 9) = 59.11, *p* < 0.001  Total gephyrin protein expression in BLA after SNAP treatment  One-way ANOVA SNAP: F (2, 9) = 0.303, *p* = 0.746 | SNO-gephyrin protein expression in BLA after SNAP treatment  Bonferroni's post hoc test  Control vs Ascorbate, *p* < 0.01  Ascorbate vs SNAP + Ascorbate, *p* < 0.001  Total gephyrin protein expression in BLA after SNAP treatment  Bonferroni's post hoc test  Control vs Ascorbate, *p* ＞ 0.05  Ascorbate vs SNAP + Ascorbate, *p* ＞ 0.05 | SNO-gephyrin protein expression in BLA after SNAP treatment  Control 0.070 ± 0.025  Ascorbate 1.000 ± 0.116  SNAP + Ascorbate 2.398 ± 0.236  Total gephyrin protein expression in BLA after SNAP treatment  Control 0.939 ± 0.116  Ascorbate 1.000 ± 0.045  SNAP + Ascorbate 0.919 ± 0.046 |
| 5B: nNOS and gephyrin protein expression  nNOS protein expression：  mPFC (n = 6)  CPu (n = 6)  vHip (n = 6)  dHip (n = 6)  CeA (n = 6)  BLA (n = 6)  Gephyrin protein expression：  mPFC (n = 6)  CPu (n = 6)  vHip (n = 6)  dHip (n = 6)  CeA (n = 6)  BLA (n = 6) |  |  | nNOS protein expression：  mPFC 1.000 ± 0.133  CPu 0.329 ± 0.021  vHip 0.449 ± 0.064  dHip 0.761 ± 0.163  CeA 0.785 ± 0.028  BLA 1.592 ± 0.134  Gephyrin protein expression：  mPFC 1.000 ± 0.028  CPu 1.117 ± 0.077  vHip 0.919 ± 0.081  dHip 0.896 ± 0.069  CeA 1.024 ± 0.159  BLA 0.899 ± 0.035 |
| 5C: SNO-gephyrin protein expression in BLA after 7-NI treatment  Control (n = 7)  Gly (n = 7)  7-NI + Gly (n = 7)  Total gephyrin protein expression in BLA after 7-NI treatment  Control (n = 7)  Gly (n = 7)  7-NI + Gly (n = 7) | SNO-gephyrin protein expression in BLA after 7-NI treatment  One-way ANOVA  7-NI: F (2, 18) = 5.354, *p* = 0.015  Total gephyrin protein expression in BLA after 7-NI treatment  One-way ANOVA  7-NI: F (2, 18) = 0.616, *p* = 0.551 | SNO-gephyrin protein expression in BLA after 7-NI treatment  Bonferroni's post hoc test  Control vs Gly, *p* < 0.05  Gly vs 7-NI + Gly, *p* < 0.05  Total gephyrin protein expression in BLA after 7-NI treatment  Bonferroni's post hoc test  Control vs Gly, *p* ＞ 0.05  Gly vs 7-NI + Gly, *p* ＞ 0.05 | SNO-gephyrin protein expression in BLA after 7-NI treatment  Control 1.000 ± 0.167  Gly 1.557 ± 0.102  Gly + 7-NI 0.977 ± 0.148  Total gephyrin protein expression in BLA after 7-NI treatment  Control 1.000 ± 0.077  Gly 0.920 ± 0.035  Gly + 7-NI 0.946 ± 0.030 |
| 5D: nNOS protein expression in BLA  LA (n = 6)  HA (n = 6) | Unpaired *t* test  *t* = 2.728, *p* < 0.05 |  | LA 1.000 ± 0.038  HA 1.364 ± 0.128 |
| 5F: nNOS protein expression in BLA  LV-shRNA-eGFP (n = 6)  LV-shRNA-nNOS (n = 5) | Unpaired *t* test  *t* = 2.296, *p* < 0.05 |  | LV-shRNA-eGFP 1.000 ± 0.126  LV-shRNA-nNOS 0.590 ± 0.124 |
| 5H: OFT  Center time:  LV-shRNA-eGFP (n = 15)  LV-shRNA-nNOS (n = 18)  Center entries:  LV-shRNA-eGFP (n = 15)  LV-shRNA-nNOS (n = 18)  Center distance:  LV-shRNA-eGFP (n = 15)  LV-shRNA-nNOS (n = 18)  Total distance:  LV-shRNA-eGFP (n = 15)  LV-shRNA-nNOS (n = 18) | Center time:  Unpaired t test  *t* = 3.165, *p* < 0.01  Center entries:  Unpaired t test  *t* = 4.243, *p* < 0.001  Center distance:  Unpaired t test  *t* = 4.634, *p* < 0.001  Total distance:  Unpaired t test  *t* = 0.602, *p* = 0.552 |  | Center time:  LV-shRNA-eGFP 13.467 ± 2.733  LV-shRNA-nNOS 25.961 ± 2.791  Center entries:  LV-shRNA-eGFP 4.867 ± 0.961  LV-shRNA-nNOS 10.111 ± 0.796  Center distance:  LV-shRNA-eGFP 0.633 ± 0.124  LV-shRNA-nNOS 1.455 ± 0.125  Total distance:  LV-shRNA-eGFP 8.771 ± 0.786  LV-shRNA-nNOS 9.339 ± 0.561 |
| 5J: EPM  Open arm duration:  LV-shRNA-eGFP (n = 15)  LV-shRNA-nNOS (n = 17)  Open arm entries:  LV-shRNA-eGFP (n = 15)  LV-shRNA-nNOS (n = 17)  Open arm distance:  LV-shRNA-eGFP (n = 15)  LV-shRNA-nNOS (n = 17)  Mean speed:  LV-shRNA-eGFP (n = 15)  LV-shRNA-nNOS (n = 17) | Open arm duration:  Unpaired t test  *t* = 3.649, *p* < 0.001  Open arm entries:  Unpaired t test  *t* = 2.056, *p* < 0.05  Open arm distance:  Unpaired t test  *t* = 3.044, *p* < 0.01  Mean speed:  Unpaired t test  *t* = 0.609, *p* = 0.547 |  | Open arm duration:  LV-shRNA-eGFP 16.727 ± 4.957  LV-shRNA-nNOS 46.047 ± 6.147  Open arm entries:  LV-shRNA-eGFP 3.733 ± 0.973  LV-shRNA-nNOS 6.882 ± 1.153  Open arm distance:  LV-shRNA-eGFP 0.410 ± 0.103  LV-shRNA-nNOS 0.842 ± 0.097  Mean speed:  LV-shRNA-eGFP 1.785 ± 0.196  LV-shRNA-nNOS 1.648 ± 0.120 |
| 5L: sGABA_A_R γ2 protein expression in BLA  LV-shRNA-eGFP (n = 14)  LV-shRNA-nNOS (n = 15) | Unpaired *t* test  *t* = 3.198, *p* < 0.01 |  | LV-shRNA-eGFP 1.000 ± 0.116  LV-shRNA-nNOS 1.730 ± 0.192 |
| 5M: tGABA_A_R γ2 protein expression in BLA  LV-shRNA-eGFP (n = 14)  LV-shRNA-nNOS (n = 15) | Unpaired *t* test  *t* = 1.076, *p* = 0.291 |  | LV-shRNA-eGFP 1.000 ± 0.055  LV-shRNA-nNOS 1.096 ± 0.069 |
| 6B: nNOS protein expression in BLA  LV-eGFP (n = 8)  LV-nNOS (n = 8) | Unpaired t test  *t* = 3.677, *p* < 0.01 |  | LV-eGFP 1.000 ± 0.042 LV-nNOS 1.185 ± 0.028 |
| 6D: SNO-gephyrin protein expression in BLA  LV-eGFP (n = 6)  LV-nNOS (n = 5)  Total gephyrin protein expression in BLA  LV-eGFP (n = 6)  LV-nNOS (n = 5) | SNO-gephyrin protein expression in BLA  Unpaired t test  *t* = 2.631, *p* < 0.05  Total gephyrin protein expression in BLA  Unpaired t test  *t* = 0.414, *p* = 0.689 |  | SNO-gephyrin protein expression in BLA  LV-eGFP 1.000 ± 0.157 LV-nNOS 1.686 ± 0.215  Total gephyrin protein expression in BLA  LV-eGFP 1.000 ± 0.079 LV-nNOS 0.961 ± 0.041 |
| 6F: OFT  Center time:  VEH (n = 10)  DETA (n = 9)  Center entries:  VEH (n = 10)  DETA (n = 9)  Center distance:  VEH (n = 10)  DETA (n = 9)  Total distance:  VEH (n = 10)  DETA (n = 9) | Center time:  Unpaired t test  *t* = 3.100, *p* < 0. 01  Center entries:  Unpaired t test  *t* = 4.115, *p* < 0.001  Center distance:  Unpaired t test  *t* = 3.103, *p* < 0. 01  Total distance:  Unpaired t test  *t* = 1.252, *p* = 0.228 |  | Center time:  VEH 28.090 ± 5.502  DETA 8.922 ± 2.219  Center entries:  VEH 8.600 ± 1.077  DETA 3.111 ± 0.735  Center distance:  VEH 1.292 ± 0.233  DETA 0.397 ± 0.160  Total distance:  VEH 8.380 ± 0.711  DETA 6.952 ± 0.907 |
| 6H: EPM  Open arm duration:  VEH (n = 10)  DETA (n = 9)  Open arm entries:  VEH (n = 10)  DETA (n = 9)  Open arm distance:  VEH (n = 10)  DETA (n = 9)  Mean speed:  VEH (n = 10)  DETA (n = 9) | Open arm duration:  Unpaired t test  *t* = 3.195, *p* < 0.01  Open arm entries:  Unpaired t test  *t* = 3.859, *p* < 0.01  Open arm distance:  Unpaired t test  *t* = 3.802, *p* < 0.01  Mean speed:  Unpaired t test  *t* = 1.908, *p* = 0.074 |  | Open arm duration:  VEH 45.020 ± 6.006  DETA 21.211 ± 4.126  Open arm entries:  VEH 4.400 ± 0.600  DETA 1.667 ± 0.333  Open arm distance:  VEH 1.256 ± 0.200  DETA 0.395 ± 0.087  Mean speed:  VEH 1.874 ± 0.303  DETA 1.169 ± 0.196 |
| 6K: OFT  Center time:  LV-eGFP + SED (n = 15)  LV-nNOS + SED (n = 14)  LV-eGFP + RUN (n = 15)  LV-nNOS + RUN (n = 15)  Center entries:  LV-eGFP + SED (n = 15)  LV-nNOS + SED (n = 14)  LV-eGFP + RUN (n = 15)  LV-nNOS + RUN (n = 15)  Center distance:  LV-eGFP + SED (n = 15)  LV-nNOS + SED (n = 14)  LV-eGFP + RUN (n = 15)  LV-nNOS + RUN (n = 15) | Center time:  Two-way ANOVA  LV-nNOS × RUN: *F* (1, 55) = 13.762, *p* < 0.001  RUN: *F* (1, 55) = 10.415, *p* < 0.01  LV-nNOS: *F* (1, 55) = 64.912, *p* < 0.001  Center entries:  LV-nNOS × RUN: *F* (1, 55) = 4.991, *p* = 0.030  RUN: *F* (1, 55) = 9.396, *p* = 0.003  LV-nNOS: *F* (1, 55) = 39.580, *p* < 0.001  Center distance:  LV-nNOS × RUN: *F* (1, 55) = 8.036, *p* = 0.006  RUN: *F* (1, 55) = 7.719, *p* = 0.007  LV-nNOS: *F* (1, 55) = 82.310, *p* < 0.001 | Center time:  Bonferroni's post hoc test  LV-eGFP + SED vs LV-eGFP + RUN, *p* < 0.001  LV-eGFP + RUN vs LV-nNOS + RUN, *p* < 0.001  Center entries:  Bonferroni's post hoc test  LV-eGFP + SED vs LV-eGFP + RUN, *p* < 0.01  LV-eGFP + RUN vs LV-nNOS + RUN, *p* < 0.001  Center distance:  Bonferroni's post hoc test  LV-eGFP + SED vs LV-eGFP + RUN, *p* < 0.01  LV-eGFP + RUN vs LV-nNOS + RUN, *p* < 0.001 | Center time:  LV-eGFP + SED 19.547 ± 1.307  LV-nNOS + SED 10.636 ± 1.440  LV-eGFP + RUN 33.767 ± 3.155  LV-nNOS + RUN 9.647 ± 1.661  Center entries:  LV-eGFP + SED 6.133 ± 0.524  LV-nNOS + SED 3.786 ± 0.547  LV-eGFP + RUN 9.200 ± 0.641  LV-nNOS + RUN 4.267 ± 0.589  Center distance:  LV-eGFP + SED 0.871 ± 0.071  LV-nNOS + SED 0.370 ± 0.058  LV-eGFP + RUN 1.322 ± 0.101  LV-nNOS + RUN 0.365 ± 0.082 |
| 6M: EPM  Open arm duration:  LV-eGFP + SED (n = 15)  LV-nNOS + SED (n = 14)  LV-eGFP + RUN (n = 14)  LV-nNOS + RUN (n = 15)  Open arm entries:  LV-eGFP + SED (n = 15)  LV-nNOS + SED (n = 14)  LV-eGFP + RUN (n = 14)  LV-nNOS + RUN (n = 15)  Open arm distance:  LV-eGFP + SED (n = 15)  LV-nNOS + SED (n = 14)  LV-eGFP + RUN (n = 14)  LV-nNOS + RUN (n = 15) | Open arm duration:  Two-way ANOVA  LV-nNOS × RUN: *F* (1, 54) = 4.070, *p* = 0.049  RUN: *F* (1, 54) = 6.005, *p* = 0.018  LV-nNOS: *F* (1, 54) = 58.060, *p* < 0.001  Open arm entries:  LV-nNOS × RUN: *F* (1, 54) = 6.084, *p* = 0.017  RUN: *F* (1, 54) = 17.570, *p* = 0.001  LV-nNOS: *F* (1, 54) = 27.310, *p* < 0.001  Open arm distance:  LV-nNOS × RUN: *F* (1, 54) = 1.100, *p* = 0.299  RUN: *F* (1, 54) = 4.401, *p* = 0.041  LV-nNOS: *F* (1, 54) = 37.540, *p* < 0.001 | Open arm duration:  Bonferroni's post hoc test  LV-eGFP + SED vs LV-eGFP + RUN, *p* < 0.05  LV-eGFP + RUN vs LV-nNOS × RUN, *p* < 0.001  Open arm entries:  Bonferroni's post hoc test  LV-eGFP + SED vs LV-eGFP + RUN, *p* < 0.001  LV-eGFP + RUN vs LV-nNOS × RUN, *p* < 0.001  Open arm distance:  Bonferroni's post hoc test  LV-eGFP + SED vs LV-eGFP + RUN, *p* = 0.181  LV-eGFP + RUN vs LV-nNOS + RUN, *p* < 0.001 | Open arm duration:  LV-eGFP + SED 60.413 ± 10.994  LV-nNOS + SED 17.893 ± 4.041  LV-eGFP + RUN 94.321 ± 9.120  LV-nNOS × RUN 21.180 ± 2.722  Open arm entries:  LV-eGFP + SED 8.133 ± 0.742  LV-nNOS + SED 5.643 ± 0.789  LV-eGFP + RUN 13.643 ± 1.234  LV-nNOS × RUN 7.200 ± 0.991  Open arm distance:  LV-eGFP + SED 1.326 ± 0.245  LV-nNOS + SED 0.378 ± 0.113  LV-eGFP + RUN 1.914 ± 0.210  LV-nNOS + RUN 0.574 ± 0.144 |
| S1A: EPM  Open arm duration:  LA (n = 16)  IA (n = 13)  HA (n = 14) | One-way ANOVA anxiety: F (2, 40) = 133.900, *p* < 0.001 | Bonferroni's post hoc test  LA vs IA, *p* < 0.001  LA vs HA, *p* < 0.001 | LA 105.706 ± 5.525  IA 42.646 ± 4.743  HA 7.807 ± 1.204 |
| S1B: EPM  Open arm entries:  LA (n = 16)  IA (n = 13)  HA (n = 14) | One-way ANOVA anxiety: F (2, 40) = 13.930, *p* < 0.001 | Bonferroni's post hoc test  LA vs IA, *p* < 0.001  LA vs HA, *p* < 0.001 | LA 13.188 ± 1.553  IA 6.539 ± 0.859  HA 4.643 ± 0.998 |
| S1C: EPM  Open arm distance:  LA (n = 16)  IA (n = 13)  HA (n = 14) | One-way ANOVA anxiety: F (2, 40) = 33.338, *p* < 0.001 | Bonferroni's post hoc test  LA vs IA, *p* < 0.001  LA vs HA, *p* < 0.001 | LA 2.461 ± 0.255  IA 1.320 ± 0.195  HA 0.245 ± 0.055 |
| S1D: EPM  Mean speed:  LA (n = 16)  IA (n = 13)  HA (n = 14) | One-way ANOVA anxiety: F (2, 40) = 0.524, *p* = 0.596 | Bonferroni's post hoc test  LA vs IA, *p* ＞ 0.05  LA vs HA, *p* ＞ 0.05 | LA 2.680 ± 0.138  IA 2.586 ± 0.135  HA 2.491 ± 0.123 |
| S2B: OFT  Center time:  VEH (n = 6)  CORT (n = 8) | Unpaired *t* test  *t* = 6.004, *p* < 0.001 |  | VEH 134.117 ± 21.023  CORT 23.638 ± 3.458 |
| S2C: OFT  Center entries:  VEH (n = 6)  CORT (n = 8) | Unpaired *t* test  *t* = 5.386, *p* < 0.001 |  | VEH 36.833 ± 3.962  CORT 12.125 ± 2.655 |
| S2D: OFT  Center distance:  VEH (n = 6)  CORT (n = 8) | Unpaired *t* test  *t* = 4.087, *p* < 0.01 |  | VEH 8.807 ± 1.809  CORT 2.001 ± 0.532 |
| S2E: OFT  Total distance:  VEH (n = 6)  CORT (n = 8) | Unpaired *t* test  *t* = 0.2978, *p* = 0.771 |  | VEH 13.168 ± 2.312  CORT 13.998 ± 1.686 |
| S2F: EPM  Open arm duration:  VEH (n = 6)  CORT (n = 7) | Unpaired *t* test  *t* = 5.821, *p* < 0.001 |  | VEH 121.567 ± 6.198  CORT 48.429 ± 10.301 |
| S2G: EPM  Open arm entries:  VEH (n = 6)  CORT (n = 7) | Unpaired *t* test  *t* = 1.323, *p* = 0.213 |  | VEH 13.667 ± 1.542  CORT 10.429 ± 1.837 |
| S2H: EPM  Open arm distance:  VEH (n = 6)  CORT (n = 7) | Unpaired *t* test  *t* = 2.868, *p* < 0.05 |  | VEH 3.598 ± 0.413  CORT 1.745 ± 0.481 |
| S2I: EPM  Mean speed:  VEH (n = 6)  CORT (n = 7) | Unpaired *t* test  *t* = 0.361, *p* = 0.725 |  | VEH 3.489 ± 0.202  CORT 3.353 ± 0.302 |
| S3A: sGABA_A_R α1 protein expression in BLA  LA (n = 4)  HA (n = 4)  tGABA_A_R α1 protein expression in BLA  LA (n = 4)  HA (n = 4) | sGABA_A_R α1 protein expression in BLA  Unpaired *t* test  *t* = 0.493, *p* = 0.639  tGABA_A_R α1 protein expression in BLA  Unpaired *t* test  *t* = 1.556, *p* = 0.171 |  | sGABA_A_R α1 protein expression in BLA  LA 1.000 ± 0.173  HA 1.154 ± 0.260  tGABA_A_R α1 protein expression in BLA  LA 1.000 ± 0.037  HA 1.139 ± 0.081 |
| S3B: sGABA_A_R α2 protein expression in BLA  LA (n = 4)  HA (n = 4)  tGABA_A_R α2 protein expression in BLA  LA (n = 4)  HA (n = 4) | sGABA_A_R α2 protein expression in BLA  Unpaired *t* test  *t* = 0.160, *p* = 0.879  tGABA_A_R α2 protein expression in BLA  Unpaired *t* test  *t* = 1.091, *p* = 0.317 |  | sGABA_A_R α2 protein expression in BLA  LA 1.000 ± 0.217  HA 0.959 ± 0.140  tGABA_A_R α2 protein expression in BLA  LA 1.000 ± 0.117  HA 1.170 ± 0.103 |
| S3C: sGABA_A_R α3 protein expression in BLA  LA (n = 6)  HA (n = 6)  tGABA_A_R α3 protein expression in BLA  LA (n = 6)  HA (n = 6) | sGABA_A_R α3 protein expression in BLA  Unpaired *t* test  *t* = 1.661, *p* = 0.128  tGABA_A_R α3 protein expression in BLA  Unpaired *t* test  *t* = 0.521, *p* = 0.614 |  | sGABA_A_R α3 protein expression in BLA  LA 1.000 ± 0.094  HA 1.200 ± 0.076  tGABA_A_R α3 protein expression in BLA  LA 1.000 ± 0.084  HA 0.931 ± 0.102 |
| S3D: sGABA_A_R β2 protein expression in BLA  LA (n = 6)  HA (n = 6)  tGABA_A_R β2 protein expression in BLA  LA (n = 6)  HA (n = 6) | sGABA_A_R β2 protein expression in BLA  Unpaired *t* test  *t* = 0.381, *p* = 0.711  tGABA_A_R β2 protein expression in BLA  Unpaired *t* test  *t* = 0.210, *p* = 0.838 |  | sGABA_A_R α3 protein expression in BLA  LA 1.000 ± 0.162  HA 0.900 ± 0.208  tGABA_A_R α3 protein expression in BLA  LA 1.000 ± 0.156  HA 1.045 ± 0.145 |
| S4B: SNO-gephyrin protein expression in BLA  VEH (n = 6)  DTT (n = 6)  Total gephyrin protein expression in BLA  VEH (n = 6)  DTT (n = 6) | SNO-gephyrin protein expression in BLA  Unpaired *t* test  *t* = 4.438, *p* < 0.01  Total gephyrin protein expression in BLA  Unpaired *t* test  *t* = 0.264, *p* = 0.797 |  | SNO-gephyrin protein expression in BLA  VEH 1.000 ± 0.143  DTT 0.320 ± 0.055  Total gephyrin protein expression in BLA  VEH 1.000 ± 0.052  DTT 0.978 ± 0.066 |
| S4D: Caspase-3 protein expression in BLA  VEH (n = 6)  DTT (n = 6)  Cleaved Caspase-3 protein expression in BLA  VEH (n = 6)  DTT (n = 6) | Caspase-3 protein expression in BLA  Unpaired *t* test  *t* = 0.212, *p* = 0.837  Cleaved Caspase-3 protein expression in BLA  Unpaired *t* test  *t* = 0.087, *p* = 0.933 |  | Caspase-3 protein expression in BLA  VEH 1.000 ± 0.077  DTT 0.964 ± 0.150  Cleaved Caspase-3 protein expression in BLA  VEH 1.000 ± 0.090  DTT 0.989 ± 0.090 |
| S4F: Bcl-2 protein expression in BLA  VEH (n = 6)  DTT (n = 6)  RIP3 protein expression in BLA  VEH (n = 6)  DTT (n = 6) | Bcl-2 protein expression in BLA  Unpaired *t* test  *t* = 0.557, *p* = 0.590  RIP3 protein expression in BLA  Unpaired *t* test  *t* = 0.168, *p* = 0.870 |  | Bcl-2 protein expression in BLA  VEH 1.000 ± 0.117  DTT 0.922 ± 0.078  RIP3 protein expression in BLA  VEH 1.000 ± 0.100  DTT 0.980 ± 0.060 |
| S4H: Caspase-1 protein expression in BLA  VEH (n = 6)  DTT (n = 6) | Unpaired *t* test  *t* = 0.884, *p* = 0.398 |  | VEH 1.000 ± 0.041  DTT 1.043 ± 0.026 |
| S5B: Gephyrin protein expression in BLA  LV-eGFP (n = 6)  LV-Gphn^C212A^ (n = 7) | Unpaired *t* test  *t* = 2.405, *p* < 0.05 |  | LV-eGFP 1.000 ± 0.120  LV-Gphn^C212A^ 1.369 ± 0.099 |
| S5C: Total gephyrin  LV-eGFP (n = 6)  LV-Gphn^C212A^ (n = 6) | Unpaired *t* test  *t* = 1.641, *p* = 0.132 |  | LV-eGFP 1.000 ± 0.109  LV-Gphn^C212A^ 0.809 ± 0.041 |
| S6B: Gephyrin protein expression in BLA  LV-eGFP (n = 4)  LV-Gphn^C284A^ (n = 5) | Unpaired *t* test  *t* = 2.566, *p* < 0.05 |  | LV-eGFP 1.000 ± 0.105  LV-Gphn^C284A^ 1.517 ± 0.158 |
| S6D: SNO-gephyrin protein expression in BLA  LV-eGFP (n = 6)  LV-Gphn^C284A^ (n = 6) | Unpaired *t* test  *t* = 4.264, *p* < 0.01 |  | LV-eGFP 1.000 ± 0.098  LV-Gphn^C284A^ 0.556 ± 0.034 |
| S6E: Total gephyrin protein expression in BLA  LV-eGFP (n = 6)  LV-Gphn^C284A^ (n = 6) | Unpaired *t* test  *t* = 1.301, *p* = 0.223 |  | LV-eGFP 1.000 ± 0.121  LV-Gphn^C284A^ 0.814 ± 0.077 |
| S6G: OFT  Center time:  LV-eGFP (n = 16)  LV-Gphn^C284A^ (n = 17)  Center entries:  LV-eGFP (n = 16)  LV-Gphn^C284A^ (n = 17)  Center distance:  LV-eGFP (n = 16)  LV-Gphn^C284A^ (n = 17)  Total distance:  LV-eGFP (n = 16)  LV-Gphn^C284A^ (n = 17) | Center time:  Unpaired *t* test  *t* = 3.354, *p* < 0.01  Center entries:  Unpaired *t* test  *t* = 3.468, *p* < 0.01  Center distance:  Unpaired *t* test  *t* = 3.854, *p* < 0.001  Total distance:  Unpaired *t* test  *t* = 0.002, *p* = 0.999 |  | Center time:  LV-eGFP 17.663 ± 2.380  LV-Gphn^C284A^ 29.353 ± 2.533  Center entries:  LV-eGFP 4.688 ± 0.631  LV-Gphn^C284A^ 8.529 ± 0.896  Center distance:  LV-eGFP 0.657 ± 0.103  LV-Gphn^C284A^ 1.218 ± 0.103  Total distance:  LV-eGFP 8.616 ± 0.677  LV-Gphn^C284A^ 8.615 ± 0.692 |
| S6I: EPM  Open arm duration:  LV-eGFP (n = 16)  LV-Gphn^C284A^ (n = 16)  Open arm entries  LV-eGFP (n = 16)  LV-Gphn^C284A^ (n = 16)  Open arm distance:  LV-eGFP (n = 16)  LV-Gphn^C284A^ (n = 16)  Mean speed:  LV-eGFP (n = 16)  LV-Gphn^C284A^ (n = 16) | Open arm duration:  Unpaired *t* test  *t* = 4.516, *p* < 0.001  Open arm entries:  Unpaired *t* test  *t* = 1.728, *p* = 0.094  Open arm distance:  Unpaired *t* test  *t* = 5.366, *p* < 0.001  Mean speed:  Unpaired *t* test  *t* = 0.331, *p* = 0.743 |  | Open arm duration:  LV-eGFP 25.244 ± 4.642  LV-Gphn^C284A^ 76.275 ± 10.303  Open arm entries:  LV-eGFP 7.938 ± 1.659  LV-Gphn^C284A^ 12.500 ± 2.054  Open arm distance:  LV-eGFP 0.375 ± 0.088  LV-Gphn^C284A^ 1.370 ± 0.163  Mean speed:  LV-eGFP 1.854 ± 0.177  LV-Gphn^C284A^ 1.950 ± 0.228 |
| S6K: sGABA_A_R γ2 protein expression in BLA  LV-eGFP (n = 6)  LV-Gphn^C284A^ (n = 6) | Unpaired *t* test  *t* = 4.693, *p* < 0.001 |  | LV-eGFP 1.000 ± 0.156  LV-Gphn^C284A^ 3.870 ± 0.591 |
| S6L: tGABA_A_R γ2 protein expression in BLA  LV-eGFP (n = 6)  LV-Gphn^C284A^ (n = 6) | Unpaired *t* test  *t* = 1.153, *p* = 0.276 |  | LV-eGFP 1.000 ± 0.041  LV-Gphn^C284A^ 0.907 ± 0.070 |
| S6N: mIPSCs amplitude LV-eGFP (n = 10)  LV-Gphn^C284A^ (n = 10) | Unpaired *t* test  *t* = 2.426, *p* < 0.05 |  | LV-eGFP 30.077 ± 2.034  LV-Gphn^C284A^ 39.560 ± 3.337 |
| S6O: mIPSCs frequency  LV-eGFP (n = 10)  LV-Gphn^C284A^ (n = 10) | Unpaired *t* test  *t* = 0.529, *p* = 0.603 |  | LV-eGFP 3.509 ± 0.356  LV-Gphn^C284A^ 3.777 ± 0.360 |
| S7B: Pal-gephyrin protein expression in BLA  LV-eGFP (n = 6)  LV-Gphn^C212A^ (n = 6)  LV-Gphn^C284A^ (n = 6) | One-way ANOVA treatment: F (2, 15) = 1.246, *p* = 0.316 | Bonferroni's post hoc test  LV-eGFP vs LV-Gphn^C212A^, *p* = 0.381  LV-eGFP vs LV-Gphn^C284A^, *p* = 0.386 | LV-eGFP 1.000 ± 0.130  LV-Gphn^C212A^ 0.800 ± 0.054  LV-Gphn^C284A^ 0.801 ± 0.110 |
| S7C: Total gephyrin protein expression in BLA  LV-eGFP (n = 6)  LV-Gphn^C212A^ (n = 6)  LV-Gphn^C284A^ (n = 6) | One-way ANOVA treatment: F (2, 15) = 0.796, *p* = 0.469 | Bonferroni's post hoc test  LV-eGFP vs LV-Gphn^C212A^, *p* ＞ 0.05  LV-eGFP vs LV-Gphn^C284A^, *p* = 0.461 | LV-eGFP 1.000 ± 0.220  LV-Gphn^C212A^ 0.890 ± 0.148  LV-Gphn^C284A^ 0.709 ± 0.105 |
| S7E: IB: GABA_A_R γ2  LV-eGFP (n = 6)  LV-Gphn^C212A^ (n = 6)  LV-Gphn^C284A^ (n = 6) | One-way ANOVA treatment: F (2, 15) = 3.014, *p* = 0.079 | Bonferroni's post hoc test  LV-eGFP vs LV-Gphn^C212A^, *p* = 0.137  LV-eGFP vs LV-Gphn^C284A^, *p* = 0.078 | LV-eGFP 1.000 ± 0.102  LV-Gphn^C212A^ 1.402 ± 0.160  LV-Gphn^C284A^ 1.463 ± 0.165 |
| S7F: Total gephyrin protein expression in BLA  LV-eGFP (n = 6)  LV-Gphn^C212A^ (n = 6)  LV-Gphn^C284A^ (n = 6) | One-way ANOVA treatment: F (2, 15) = 0.031, *p* = 0.969 | Bonferroni's post hoc test  LV-eGFP vs LV-Gphn^C212A^, *p* ＞ 0.05  LV-eGFP vs LV-Gphn^C284A^, *p* ＞ 0.05 | LV-eGFP 1.000 ± 0.049  LV-Gphn^C212A^ 0.987 ± 0.081  LV-Gphn^C284A^ 1.016 ± 0.103 |
| S8A: nNOS mRNA expression in BLA  LA (n = 13)  HA (n = 14) | Unpaired *t* test  *t* = 2.357, *p* < 0.05 |  | LA 1.000 ± 0.067  HA 1.292 ± 0.102 |
| S8B: eNOS mRNA expression in BLA  LA (n = 14)  HA (n = 15) | Unpaired *t* test  *t* = 0.399, *p* = 0.693 |  | LA 1.000 ± 0.116  HA 0.940 ± 0.097 |
| S8C: iNOS protein expression in BLA  LA (n = 7)  HA (n = 7) | Unpaired *t* test  *t* = 0.961, *p* = 0.355 |  | LA 1.000 ± 0.096  HA 1.140 ± 0.109 |
| S8D: nNOS protein expression in BLA  SED (n = 11)  RUN (n = 10) | Unpaired *t* test  *t* = 2.942, *p* < 0.01 |  | SED 1.000 ± 0.089  RUN 0.668 ± 0.067 |
| S8F: OFT  Center time:  VEH (n = 9)  KT5823 (n = 10)  Center entries:  VEH (n = 9)  KT5823 (n = 10)  Center distance:  VEH (n = 9)  KT5823 (n = 10)  Total distance:  VEH (n = 9)  KT5823 (n = 10) | Center time:  Unpaired *t* test  *t* = 0.554, *p* = 0.587  Center entries:  Unpaired *t* test  *t* = 0.172, *p* = 0.865  Center distance:  Unpaired *t* test  *t* = 0.457, *p* = 0.653  Total distance:  Unpaired *t* test  *t* = 1.057, *p* = 0.306 |  | Center time:  VEH 8.422 ± 1.329  KT5823 10.120 ± 2.646  Center entries:  VEH 3.222 ± 0.741  KT5823 3.400 ± 0.718  Center distance:  VEH 0.455 ± 0.079  KT5823 0.384 ± 0.130  Total distance:  VEH 8.373 ± 0.680  KT5823 7.483 ± 0.515 |
| S8H: EPM  Open arm duration:  VEH (n = 9)  KT5823 (n = 10)  Open arm entries:  VEH (n = 9)  KT5823 (n = 10)  Open arm distance:  VEH (n = 9)  KT5823 (n = 10)  Mean speed:  VEH (n = 9)  KT5823 (n = 10) | Open arm duration:  Unpaired *t* test  *t* = 1.240, *p* = 0.232  Open arm entries:  Unpaired *t* test  *t* = 0.078, *p* = 0.939  Open arm distance:  Unpaired *t* test  *t* = 0.311, *p* = 0.760  Mean speed:  Unpaired *t* test  *t* = 0.095, *p* = 0.926 |  | Open arm duration:  VEH 40.022 ± 11.910  KT5823 23.690 ± 6.458  Open arm entries:  VEH 3.667 ± 0.500  KT5823 3.600 ± 0.670  Open arm distance:  VEH 0.615 ± 0.185  KT5823 0.707 ± 0.227  Mean speed:  VEH 1.478 ± 0.248  KT5823 1.445 ± 0.241 |
| S9C: SNO-gephyrin protein expression in BLA  LV-shRNA-eGFP (n = 7)  LV-shRNA-nNOS (n = 6) | Unpaired *t* test  *t* = 2.660, *p* < 0.05 |  | LV-shRNA-eGFP 1.000 ± 0.122  LV-shRNA-nNOS 0.606 ± 0.071 |
| S9D: Total gephyrin protein expression in BLA  LV-shRNA-eGFP (n = 7)  LV-shRNA-nNOS (n = 6) | Unpaired *t* test  *t* = 0.305, *p* = 0.766 |  | LV-shRNA-eGFP 1.000 ± 0.096  LV-shRNA-nNOS 0.957 ± 0.106 |
| S9F: OFT  Center time:  VEH (n = 10)  7-NI (n = 10)  Center entries:  VEH (n = 10)  7-NI (n = 10)  Center distance:  VEH (n = 10)  7-NI (n = 10)  Total distance:  VEH (n = 10)  7-NI (n = 10) | Center time:  Unpaired t test  *t* = 3.043, *p* < 0. 01  Center entries:  Unpaired t test  *t* = 3.604, *p* < 0.01  Center distance:  Unpaired t test  *t* = 4.418, *p* < 0.001  Total distance:  Unpaired t test  *t* = 0.237, *p* = 0.816 |  | Center time:  VEH 4.630 ± 1.939  7-NI 18.370 ± 4.078  Center entries:  VEH 2.300 ± 0.651  7-NI 6.400 ± 0.933  Center distance:  VEH 0.237 ± 0.079  7-NI 0.980 ± 0.148  Total distance:  VEH 7.941 ± 0.607  7-NI 8.165 ± 0.724 |
| S9H: EPM  Open arm duration:  VEH (n = 16)  7-NI (n = 20)  Open arm entries:  VEH (n = 16)  7-NI (n = 20)  Open arm distance:  VEH (n = 16)  7-NI (n = 20)  Mean speed:  VEH (n = 16)  7-NI (n = 20) | Open arm duration:  Unpaired t test  *t* = 4.060, *p* < 0.001  Open arm entries:  Unpaired t test  *t* = 2.750, *p* < 0.01  Open arm distance:  Unpaired t test  *t* = 3.419, *p* < 0.01  Mean speed:  Unpaired t test  *t* = 0.002, *p* = 0.998 |  | Open arm duration:  VEH 7.100 ± 1.781  7-NI 29.750 ± 4.769  Open arm entries:  VEH 2.438 ± 0.398  7-NI 4.750 ± 0.680  Open arm distance:  VEH 0.171 ± 0.041  7-NI 0.719 ± 0.139  Mean speed:  VEH 1.725 ± 0.161  7-NI 1.725 ± 0.219 |
| S9J: sGABA_A_R γ2 protein expression in BLA  VEH (n = 7)  7-NI (n = 8) | Unpaired *t* test  *t* = 2.304, *p* < 0.05 |  | VEH 1.000 ± 0.179  7-NI 1.881 ± 0.321 |
| S9K: tGABA_A_R γ2 protein expression in BLA  VEH (n = 7)  7-NI (n = 8) | tGABA_A_R γ2 protein expression in BLA  Unpaired *t* test  *t* = 1.467, *p* = 0.166 |  | tGABA_A_R γ2 protein expression in BLA  VEH 1.000 ± 0.092  7-NI 1.198 ± 0.097 |
| S10D: OFT  Center time:  LV-eGFP (n = 9)  LV-nNOS (n = 9)  Center entries:  LV-eGFP (n = 9)  LV-nNOS (n = 9)  Center distance:  LV-eGFP (n = 9)  LV-nNOS (n = 9)  Total distance:  LV-eGFP (n = 9)  LV-nNOS (n = 9) | Center time:  Unpaired t test  *t* = 2.737, *p* < 0.05  Center entries:  Unpaired t test  *t* = 2.696, *p* < 0.05  Center distance:  Unpaired t test  *t* = 2.788, *p* < 0.05  Total distance:  Unpaired t test  *t* = 1.339, *p* = 0.199 |  | Center time:  LV-eGFP 27.222 ± 4.652  LV-nNOS 13.311 ± 2.046  Center entries:  LV-eGFP 8.333 ± 1.546  LV-nNOS 4.000 ± 0.441  Center distance:  LV-eGFP 1.052 ± 0.144  LV-nNOS 0.580 ± 0.088  Total distance:  LV-eGFP 6.862 ± 0.633  LV-nNOS 8.067 ± 0.640 |
| S10F: EPM  Open arm duration:  LV-eGFP (n = 11)  LV-nNOS (n = 11)  Open arm entries:  LV-eGFP (n = 11)  LV-nNOS (n = 11)  Open arm distance:  LV-eGFP (n = 11)  LV-nNOS (n = 11)  Mean speed:  LV-eGFP (n = 11)  LV-nNOS (n = 11) | Open arm duration:  Unpaired t test  *t* = 2.714, *p* < 0.05  Open arm entries:  Unpaired t test  *t* = 0.975, *p* = 0.341  Open arm distance:  Unpaired t test  *t* = 2.591, *p* < 0.05  Mean speed:  Unpaired t test  *t* = 0.094, *p* = 0.926 |  | Open arm duration:  LV-eGFP 57.336 ± 8.735  LV-nNOS 27.991 ± 6.375  Open arm entries:  LV-eGFP 8.455 ± 1.171  LV-nNOS 6.727 ± 1.329  Open arm distance:  LV-eGFP 1.183 ± 0.228  LV-nNOS 0.510 ± 0.124  Mean speed:  LV-eGFP 2.091 ± 0.183  LV-nNOS 2.124 ± 0.305 |
| S10H: sGABA_A_R γ2 protein expression in BLA  LV-eGFP (n = 9)  LV-nNOS (n = 9) | Unpaired *t* test  *t* = 2.102, *p* = 0.052 |  | LV-eGFP 1.000 ± 0.149  LV-nNOS 0.626 ± 0.097 |
| S10I: tGABA_A_R γ2 protein expression in BLA  LV-eGFP (n = 9)  LV-nNOS (n = 9) | Unpaired *t* test  *t* = 1.677, *p* = 0.113 |  | LV-eGFP 1.000 ± 0.055  LV-nNOS 1.153 ± 0.073 |
| S10K: sGABA_A_R γ2 protein expression in BLA  VEH (n = 10)  DETA (n = 10) | Unpaired *t* test  *t* = 3.438, *p* < 0.01 |  | VEH 1.000 ± 0.124  DETA 0.508 ± 0.072 |
| S10L: tGABA_A_R γ2 protein expression in BLA  VEH (n = 10)  DETA (n = 10) | Unpaired *t* test  *t* = 0.735, *p* = 0.472 |  | VEH 1.000 ± 0.060  DETA 0.940 ± 0.056 |
| S10M: OFT  Total distance:  LV-eGFP + SED (n = 15)  LV-nNOS + SED (n = 14)  LV-eGFP + RUN (n = 15)  LV-nNOS + RUN (n = 15) | Two-way ANOVA  LV-nNOS × RUN: *F* (1, 55) = 0.058, *p* = 0.811  RUN: *F* (1, 55) = 3.281, *p* = 0.076  LV-nNOS: *F* (1, 55) = 0.282, *p* = 0.598 | Bonferroni's post hoc test  LV-eGFP + SED vs LV-eGFP + RUN, *p* > 0.05  LV-eGFP + RUN vs LV-nNOS + RUN, *p* > 0.05 | LV-eGFP + SED 6.766 ± 0.696  LV-nNOS + SED 6.326 ± 0.575  LV-eGFP + RUN 7.662 ± 0.576  LV-nNOS + RUN 7.497 ± 0.391 |
| S10N: EPM  Mean speed:  LV-eGFP + SED (n = 15)  LV-nNOS + SED (n = 14)  LV-eGFP + RUN (n = 14)  LV-nNOS + RUN (n = 15) | Two-way ANOVA  LV-nNOS × RUN: *F* (1, 54) = 1.208, *p* = 0.277  RUN: *F* (1, 54) = 1.321, *p* = 0.255  LV-nNOS: *F* (1, 54) = 7.769, *p* = 0.007 | Bonferroni's post hoc test  LV-eGFP + SED vs LV-eGFP + RUN, *p* > 0.05  LV-eGFP + RUN vs LV-nNOS + RUN, *p* < 0.05 | LV-eGFP + SED 2.020 ± 0.206  LV-nNOS + SED 1.703 ± 0.174  LV-eGFP + RUN 2.443 ± 0.186  LV-nNOS + RUN 1.712 ± 0.182 |
